# Thermally Programmable Two‐Port Non‐Hermitian Acoustic Metastructure for Broadband and Direction‐Dependent Absorption

**DOI:** 10.1002/advs.202524228

**Published:** 2026-01-28

**Authors:** Zichao Guo, Zhendong Li, Ziping Lei, Zirui Yang, Kexin Zeng, Zheng Fan, Zhonggang Wang

**Affiliations:** ^1^ School of Traffic & Transportation Engineering Central South University Changsha Hunan China; ^2^ School of Mechanical and Aerospace Engineering Nanyang Technological University Singapore Singapore; ^3^ The State Key Laboratory of Heavy‐duty and Express High‐power Electric Locomotive Changsha Hunan China; ^4^ Department of Mechanical Engineering National University of Singapore Singapore Singapore

**Keywords:** acoustic metastructure, asymmetric absorption, exceptional point, thermal modulation

## Abstract

A thermally programmable two‐port non‐Hermitian acoustic metastructure is proposed to realize broadband and direction‐dependent sound absorption by exploiting temperature as a non‐geometric air‐property tuning variable. Within a unified transfer‐matrix and electro‐acoustic circuit modeling framework, the influence of thermal variation in air density, viscosity, and speed of sound on impedance matching and loss–leakage coupling is quantitatively described. The model reveals that thermal modulation shifts the scattering‐matrix eigenvalues and adjusts the critical‐coupling condition, enabling asymmetric absorption associated with exceptional point behavior. Numerical analyses show that, under thermally induced parameter changes, the structure achieves an effective bandwidth of 321 Hz at a deep subwavelength scale, while maintaining robust one‐sided suppression of reflection. Temperature‐driven transitions between under‐damped, critically damped, and over‐damped states further demonstrate how non‐Hermitian coupling can be programmably controlled without any geometric modification. The proposed framework consolidates theoretical modeling and numerical prediction, clarifies the mechanism of thermally programmable asymmetric absorption, and provides a compact route for broadband sound control in extreme or variable‐temperature environments. These results offer fundamental guidance for designing programmable acoustic absorbers and establish a foundation for future material and high‐temperature implementations.

## Graphical abstract

A thermally programmable two‐port non‐Hermitian acoustic metastructure is presented, where temperature acts as a non‐geometric tuning variable. Thermal modulation reconfigures impedance matching and loss‐leakage coupling, enabling exceptional‐point driven, broadband, and direction‐dependent sound absorption without requiring geometric reconfiguration.

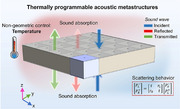

## 1 Introduction

High‐temperature and noise‐intensive environments, such as those encountered in aerospace propulsion systems, high‐speed railway braking systems, and industrial equipment, impose stringent demands on acoustic structures used for noise and energy control. In these extreme operating conditions, strong acoustic excitation often coexists with significant thermal effects, giving rise to thermo‐acoustic interactions that alter air properties and compromise the performance of conventional absorbers. Achieving efficient sound absorption at low frequencies is particularly challenging because of the long acoustic wavelength and inherently weak viscous losses of air, which usually require bulky resonant cavities or multilayer configurations [1, 2, 3, 4]. Therefore, it is essential to develop structural designs that can maintain stable and broadband acoustic functionality under elevated‐temperature environments, without relying solely on temperature‐resistant materials or intricate geometries.

Over the past decade, numerous strategies have been explored to enhance the sound‐absorbing capability of engineered structures. Geometrically tuned absorbers such as Helmholtz resonators [5, 6], coiled channels [7, 8], and porous composites [9, 10, 11] have achieved subwavelength performance by optimizing structural parameters. Meanwhile, acoustic metamaterials and metastructures have provided new physical insights through local resonances, wave‐structure coupling, and impedance‐matching design [12, 13, 14]. However, despite these achievements, most existing absorbers rely on fixed geometries and temperature‐independent parameters, making their performance highly sensitive to thermal variations. The intrinsic temperature dependence of air properties, including density, viscosity, and sound speed, often disrupts impedance matching and shifts resonance frequencies, leading to performance degradation. Moreover, high‐temperature effects are typically regarded as detrimental disturbances rather than controllable design variables, leaving the interaction between heat and sound largely underexplored. As a result, the fundamental trade‐off between temperature‐dependent performance consistency and broadband acoustic efficiency remains a persistent challenge.

Recent research efforts have focused on understanding the acoustic behavior of engineered absorbers under varying thermal environments, particularly in high‐temperature conditions relevant to aerospace, transportation, and industrial applications [15, 16, 17]. These studies have provided valuable insights into how temperature influences sound absorption mechanisms in porous, perforated, and architected structures, clarifying the roles of air‐property variation, impedance matching, and resonance shift at elevated temperatures [18, 19]. By systematically examining the thermal sensitivity of different absorber configurations, these works have established a foundation for high‐temperature acoustic modeling and experimental characterization. Nevertheless, in most of these studies, temperature is treated as an environmental factor that passively affects acoustic performance, rather than as a controllable design variable that can be intentionally utilized to tailor energy dissipation or coupling behavior.

Although previous research has examined the influence of temperature on acoustic performance, it is worth noting that temperature directly affects viscous dissipation and impedance matching—two key factors governing the balance between intrinsic loss and external leakage. This connection naturally links thermal modulation to the physics of non‐Hermitian acoustic systems, where engineered asymmetric losses can give rise to exceptional point (EP) behavior. Here, an EP corresponds to the coalescence of both the eigenvalues and eigenvectors of the system, enabling precise control over energy transport and critical coupling [20, 21].

In non‐Hermitian acoustics, loss engineering and structural asymmetry have been widely employed to achieve unidirectional absorption and asymmetric reflection [22, 23, 24]. However, most of these approaches rely on geometric tuning or the incorporation of lossy materials. The role of temperature in modulating loss asymmetry and EP evolution has yet to be systematically explored. Temperature has rarely been considered as a programmable variable capable of actively regulating non‐Hermitian coupling strength and shifting the location of EPs.

Building upon this understanding, the present work incorporates temperature directly into the damping and coupling design framework of acoustic metastructures, treating it as a programmable non‐geometric parameter for controllable modulation of intrinsic losses and impedance matching within a two‐port system. When a temperature gradient is applied across the system, the coupled resonators are expected to exhibit direction‐dependent responses, giving rise to functional asymmetry without altering the overall geometry. While such direction‐dependent acoustic responses have been successfully realized within the framework of acoustic bianisotropy through carefully designed geometric asymmetry or material contrast in previous studies [25, 26], the present work demonstrates that comparable effective asymmetry can be continuously tuned by temperature as a non‐geometric control parameter, without modifying the underlying structural geometry.

This study develops a theoretical and numerical framework for thermally programmable acoustic metastructures (TPAM), revealing direction‐dependent and broadband absorption without geometric modification. The TPAM establishes a thermal‐acoustic coupling framework, wherein temperature modulates the loss distribution and impedance balance, effectively transforming thermal effects from an environmental constraint into a design variable. The structure demonstrates robust and thermally reinforced acoustic performance, achieving broadband absorption with an effective bandwidth of 321 Hz at a deeply subwavelength scale. This design ensures strong low‐frequency absorption and compact configuration while maintaining excellent tunability, manufacturability, and practical adaptability. Overall, the proposed strategy bridges temperature‐dependent physical mechanisms with engineering realization, offering a non‐geometric design paradigm for multifunctional acoustic composites capable of stable and programmable operation in extreme thermal environments.

## 2 Design Framework

**FIGURE 1:**
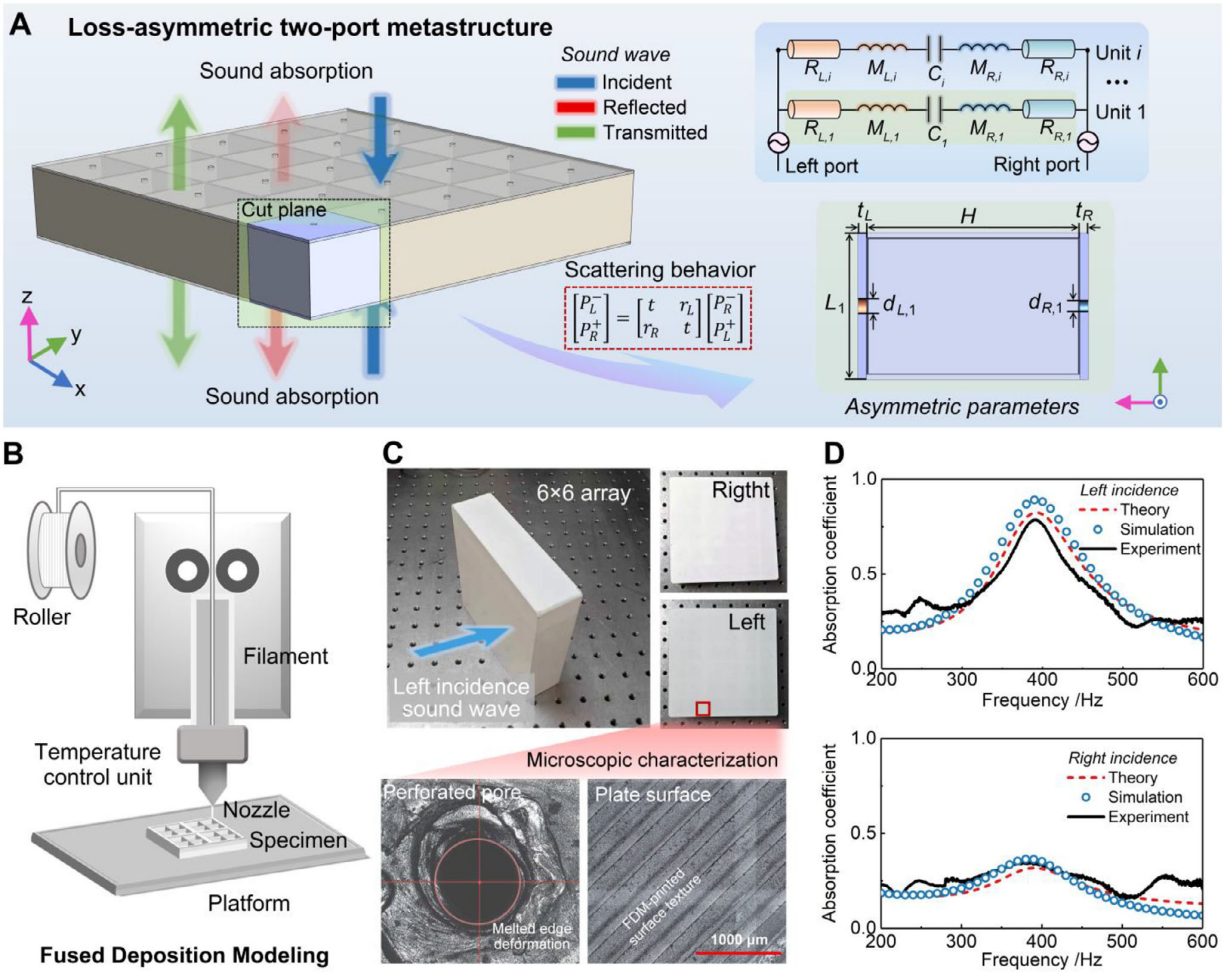
Conceptual schematic and equivalent circuit model of the loss‐asymmetric TPAM. (A) Conceptual schematic and equivalent circuit model of the loss‐asymmetric TPAM, illustrating its directional scattering characteristics and geometric parameters. (B) Schematic of the FDM printing process used for sample fabrication. (C) Fabricated TPAM samples, together with microscopic images of the plate surface and perforated pores, demonstrating good geometric fidelity with minor fabrication‐induced irregularities. (D) Experimental validation of the asymmetric absorption behavior under normal‐incidence conditions from two sides.

Figure 1 presents the conceptual design and theoretical framework of the proposed loss‐asymmetric two‐port acoustic system, referred to as the TPAM. The system consists of two parallel perforated plates separated by a cavity of thickness *H*. Although the cavity length *L* and plate thicknesses *t* are identical on both sides, structural asymmetry is introduced through different pore diameters on the left and right plates (*d*_*L*,1_,  *d*_*R*,1_​). This geometric difference gives rise to unequal viscous and thermal boundary‐layer losses, thereby establishing a loss‐asymmetric acoustic channel. The asymmetric loss distribution between the two ports renders the structure a passive non‐Hermitian system, where the imbalance of dissipation channels leads to non‐unitary scattering, while preserving reciprocity [22, 23, 24]. To quantitatively describe this property, the scattering behavior of the TPAM is formulated using a two‐port transfer‐matrix representation

(1) $$\;\left[\begin{array}{c} P_{L}^{-}\\ P_{R}^{+} \end{array}\right]=\left[\begin{array}{cc} t & r_{L}\\ r_{R} & t \end{array}\right]\;\left[\begin{array}{c} P_{R}^{-}\\ P_{L}^{+} \end{array}\right]$$

where *r_L_* and *r_R_* ​ denote the reflection coefficients for left and right incidence, and *t* is the transmission coefficient. For an ideally balanced geometry, the absorption response is symmetric, whereas asymmetric loss distributions lead to direction‐dependent absorption. The absorption coefficients for left‐ and right‐side incidence can be expressed respectively as: α_*L*_ =  1 − |*r_L_* |^2^ − |*t*|^2^, α_*R*_ =  1 − |*r_R_* |^2^ − |*t*|^2^. To theoretically characterize the acoustic characteristics of the system, the transfer matrix method (TMM) is employed. Detailed derivations of the analytical model are provided in the Sections S1 and S2.

For a more intuitive interpretation of the underlying physics, the acoustic system is mapped onto an electro‐acoustic equivalent RLC circuit model, also shown in Figure 1A. Each perforated plate is modeled by a resistance representing viscous and thermal dissipation and an inductance associated with the inertial mass of the perforations, while the intermediate cavity is characterized by a compliance corresponding to the acoustic compressibility. Asymmetry in pore size directly translates to unbalanced resistive elements (*R*_*L*,*i*_,*R*_*R*,*i*_), reflecting asymmetric viscous and thermal losses and the inductive terms (*L*_*L*,*i*_,*L*_*R*,*i*_) associated with the inertial mass of the perforations. The circuit analogy highlights that, due to the unbalanced resistive and inductive elements, the TPAM behaves as a passive non‐Hermitian system with engineered asymmetric losses. The equivalent‐circuit description reveals a clear link between geometry and acoustic response. Structural asymmetry governs reflection, transmission, and absorption, thereby producing pronounced absorption asymmetry.

Based on this analytical model, a prototype of the designed TPAM with asymmetric loss was fabricated and experimentally tested under room‐temperature conditions (293 K), confirming the asymmetric absorption behavior predicted by the analytical model. 3D printing has emerged as an efficient manufacturing technique for fabricating complex structures with high geometric freedom [6, 27, 28]. In this work, the TPAM prototype was fabricated using fused deposition modeling (FDM), as shown in Figure 1B, in which thermoplastic filaments are heated and extruded through a nozzle to form each layer according to predesigned tool paths. Among various additive manufacturing methods, FDM was selected because of its simplicity, low cost, and excellent compatibility with engineering polymers, making it well suited for rapid prototyping of acoustic components [29, 30]. To further evaluate the fabrication quality, microscopic observations were carried out on the printed plate surfaces and perforated regions using an optical microscope. As shown in Figure 1C, the printed sample exhibits well‐formed perforations and smooth plate surfaces, confirming that the overall geometry is accurately reproduced by the FDM process [12]. Minor traces of melted edges and surface texture are visible around some perforations, which are inherent to the FDM printing process, but they do not significantly affect the overall dimensional accuracy or structural integrity. These observations verify that the fabricated metastructure maintains good geometric fidelity and is suitable for subsequent acoustic characterization.

Figure 1D compares the sound absorption spectra of the proposed TPAM, as obtained from theoretical predictions, numerical simulations, and experimental measurements under left‐ and right‐side incidence. The simulations were performed in COMSOL Multiphysics using the pressure acoustics and thermoviscous acoustics modules to model the incident sound field and the viscous–thermal losses within the subwavelength pores, respectively. The experimental measurements of sound absorption were performed in a custom‐built rectangular impedance tube following the ASTM E2611‐17 standard [31], detailed descriptions are provided in Section S3 [32]. The results clearly exhibit asymmetric absorption behavior, consistent with both the theoretical and simulated results. Under left incidence, the structure shows an obvious absorption peak at approximately 394 Hz, where the absorption indicates highly efficient energy dissipation. In contrast, for right incidence, a weaker absorption peak appears near 370 Hz with a maximum value around 0.3, confirming the existence of asymmetric absorption characteristics. The overall agreement between the experimental, simulated, and theoretical results validates the accuracy of the analytical model and the fidelity of the fabricated sample. Microscopic observations reveal slight edge melting around the perforations and minor surface roughness on the printed plates. These fabrication imperfections slightly alter the effective pore geometry and increase local viscous losses [33, 34], which may account for the small deviations between the simulated and experimental absorption results.

## 3 Asymmetric Absorption under Varying Temperatures

**FIGURE 2:**
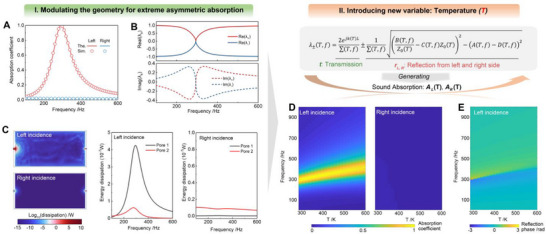
Asymmetric and thermally programmable sound absorption in a passive non‐Hermitian TPAM. (A) Theoretical and simulated absorption spectra showing nearly perfect absorption from one direction and negligible absorption from the opposite side. (B) Complex eigenvalue distributions revealing the EP at which the eigenvalues coalesce, enabling extreme asymmetric absorption. (C) Spatial and spectral energy‐dissipation profiles illustrating strong viscous loss for left incidence and minimal dissipation for right incidence. (D) Temperature‐dependent absorption maps indicating that increasing temperature shifts and broadens the absorption band while preserving directional asymmetry. (E) Phase evolution of the reflected wave demonstrating thermally induced modulation of the complex reflection coefficient.

Following the experimental validation in Figure 1, which demonstrates the occurrence of asymmetric absorption, Figure 2 further highlights the extreme performance achievable in such a loss‐asymmetric system. Figure 2A presents the theoretical and numerical absorption spectra for incidence from the left and right sides under room temperature conditions. The results reveal an extreme absorption asymmetry: for left incidence, the absorption coefficient reaches nearly perfect absorption around 295 Hz, whereas for right incidence it remains close to zero over the entire frequency range. This striking contrast demonstrates that a purely passive structure can produce strongly directional absorption by engineering imbalanced loss alone—without introducing active elements or gain materials [20]. The geometric parameters are listed in Section S4. The theoretical predictions based on TMM agree very well with numerical simulations, confirming that asymmetric loss is the dominant mechanism governing the observed behavior.

The system is modeled as a two‐port non‐Hermitian system, in which temperature acts as a programmable parameter that modifies the effective density, viscosity, and sound speed of the surrounding medium. This treatment enables a unified description of both geometric and thermal modulation within the same theoretical framework. To provide deeper insight into the physics of this phenomenon, Figure 2B plots the complex eigenvalues λ_±_ of the system as a function of frequency. The eigenvalues, which are modulated by temperature (*T*), are obtained by solving the characteristic equation of the two‐port scattering matrix, which can be analytically expressed as:

(2) $$\begin{array}{cc} & \lambda_{\pm }\;\left(T,f\right)\\ & \;=\frac{2e^{jk\left(T\right)\left(H+t_{L}+t_{R}\right)}}{\sum \left(T,f\right)}\;\\ & \;\pm \frac{1}{\sum \left(T,f\right)}\sqrt{{\left(\frac{B\left(T,f\right)}{Z_{0}\left(T\right)}-C\left(T,f\right)Z_{0}\left(T\right)\right)}^{2}-{\left(A\left(T,f\right)-D\left(T,f\right)\right)}^{2}} \end{array}$$

where

(3) $$\;\sum \left(T,f\right)=A\left(T,f\right)+\frac{B\left(T,f\right)}{Z_{0}\left(T\right)}+C\left(T,f\right)Z_{0}\left(T\right)+D\left(T,f\right)$$

Here, *A*(*T*, *f*), *B*(*T*, *f*), *C*(*T*, *f*), and *D*(*T*, *f*) represent the temperature‐ and frequency‐dependent elements of the transfer matrix for the two‐port system [35], *Z*_0_(*T*) is the characteristic impedance of air, and *k*(*T*) denotes the temperature‐dependent wavenumber. This formulation explicitly integrates geometric asymmetry and thermal modulation, enabling direct evaluation of how temperature variations influence the eigenvalue trajectories and coupling behavior. Detailed descriptions are provided in Section S2. Figure 2B shows that the real and imaginary parts of the two eigenvalues coalesce at the same frequency, corresponding to the EP, at which the real and imaginary parts of the two eigenvalues coalesce at the same frequency [36, 37, 38]. Operating near the EP enables the realization of extreme one‐sided absorption: energy incident from one direction is almost completely dissipated, while that from the opposite direction is nearly reflected. The emergence of this EP thus provides the fundamental physical origin of the observed asymmetric absorption in the passive non‐Hermitian system.

Further clarification of the loss mechanism is provided in Figure 2C, which presents the spatial distribution of acoustic energy dissipation at the resonance frequency and compares the dissipated power under left and right incidence across the full frequency range. For left incidence, the dissipation field is strongly concentrated near the entrance‐side perforation and cavity interface, where the viscous boundary layers are most active, giving rise to nearly perfect absorption. In contrast, for right incidence, only negligible dissipation occurs throughout the structure, and most of the incident energy is reflected back into the surrounding medium. The quantitative comparison of the dissipated power at the two perforated pores confirms that the loss‐asymmetric design leads to an order‐of‐magnitude difference in viscous dissipation between the two sides. These results verify that the observed asymmetric absorption arises from the engineered asymmetric loss distribution.

Building on this foundation, Figure 2D,E introduce temperature as an additional programmable variable that modulates the acoustic response of the system. Because the viscous and thermal boundary‐layer properties of air are temperature‐dependent, varying temperature effectively alters both the intrinsic loss coefficients and the effective impedance contrast between the two ports. The corresponding temperature‐dependent eigenvalue trajectories, exhibited in the Section S5, further confirm this modulation mechanism by showing the evolution of modal damping with temperature. As the temperature increases, the left‐incidence absorption band gradually broadens and shifts toward higher frequencies, as shown in Figure 2D, whereas the right‐incidence response remains nearly unchanged. This behavior confirms that temperature serves as an efficient external parameter for continuously tuning the degree of loss asymmetry without modifying the structural geometry. The corresponding phase evolution of the reflected wave (as shown in Figure 2E) further illustrates that thermal modulation can dynamically reshape the complex reflection coefficient, paving the way for broadband and thermally controllable asymmetric absorption discussed in the next section.

## 4 Thermal Modulation of Absorption Performance

Thermal modulation provides an effective route to dynamically tune the intrinsic damping behavior and absorption performance of the loss‐asymmetric TPAM. Unlike purely geometric optimization, temperature variation simultaneously alters the viscous‐thermal properties of the background medium, such as air viscosity, density, and sound speed, thereby modifying both the resistive and reactive components of the acoustic impedance. This dual influence enables controllable modulation of over‐damped and under‐damped regimes and directly affects the bandwidth and efficiency of sound absorption. In this section, we first analyze the damping states of a single unit and examine how temperature variation reshapes these damping conditions, and finally demonstrate how the interplay between distinct damping states leads to broadband absorption in coupled configurations.

### 4.1 Temperature‐Dependent Damping Transitions

**FIGURE 3:**
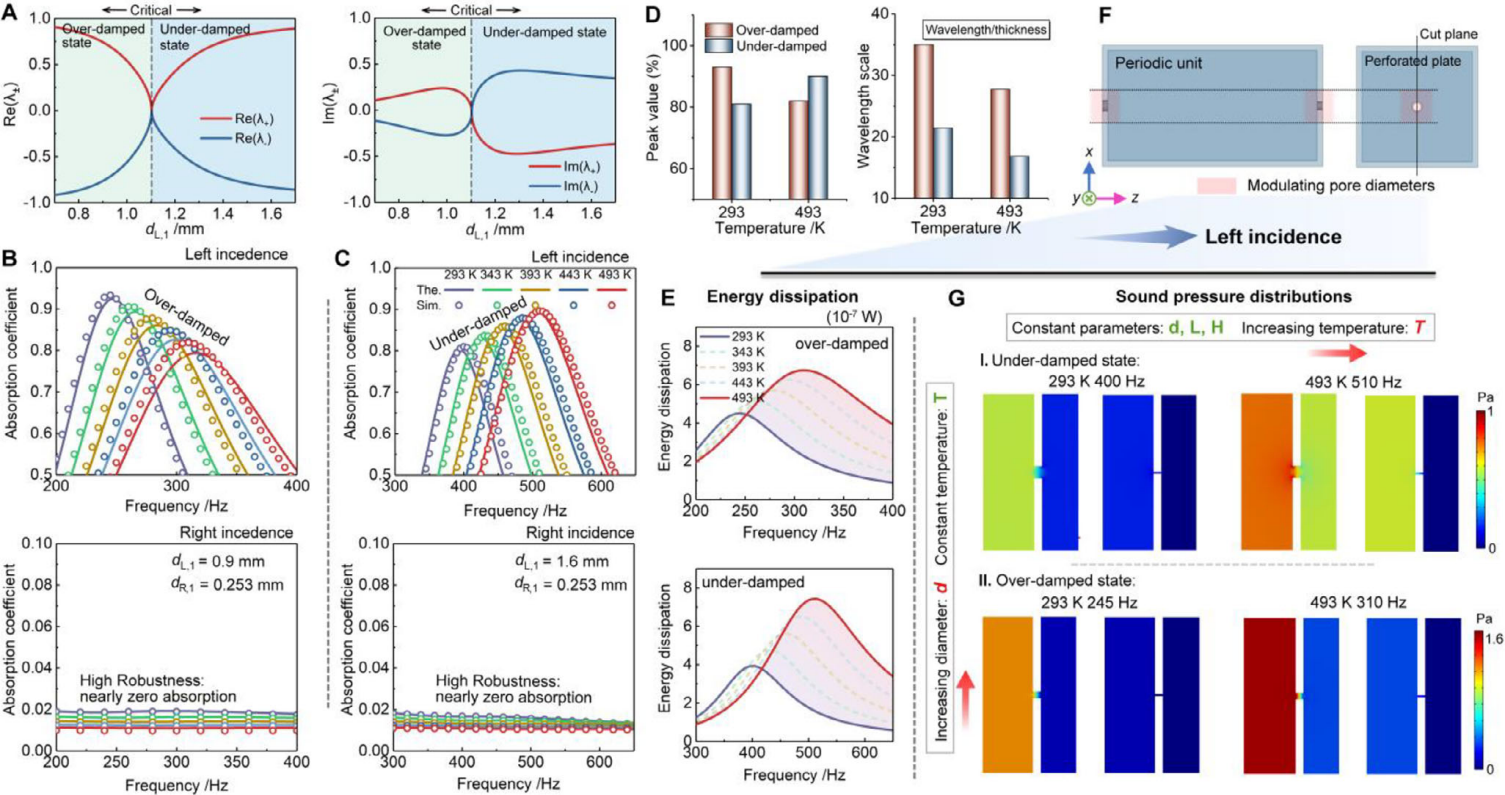
Temperature‐dependent damping transitions and energy dissipation characteristics of the loss‐asymmetric TPAM. (A) Evolution of the real and imaginary parts of the eigenvalues as a function of the left‐side pore diameter *d*_L,1_ under room‐temperature conditions, revealing the transition from the over‐damped to under‐damped state at the critical coupling condition. (B,C) Simulated and theoretical absorption spectra of the over‐damped and under‐damped configurations at different temperatures. (D) Comparison of peak absorption values and wavelength‐to‐thickness ratios at 293 and 493 K for both damping states. (E) Energy dissipation for the two damping states, highlighting the evolution of absorption and total dissipated power due to varying loss‐leakage balance. (F) Schematic of the periodic unit cell with fixed geometry and variable pore diameters, excited by left‐side incidence. (G) Sound pressure field distributions for under‐damped and over‐damped states at 293 and 493 K, demonstrating that temperature‐induced variations in pressure contrast govern the transition between weakly and strongly coupled resonance modes.

To elucidate the role of damping in shaping asymmetric absorption, the analysis first exhibits the eigenvalue characteristics at room temperature and then investigates the manner in which temperature variations modify the system response. Figure 3A shows the evolution of the real and imaginary parts of the eigenvalues with respect to the left‐side pore diameter *d*_L,1_ under room‐temperature conditions. At the EP, the system reaches a critical balance between intrinsic loss and external leakage, signifying the transition from an over‐damped to an under‐damped state [12, 21, 39]. Here, the critical coupling condition refers to the situation in which the intrinsic loss rate is equal to the external radiation leakage rate, resulting in maximized energy absorption. In the over‐damped state, internal loss dominates external leakage, resulting in broadband but inefficient absorption. In contrast, the under‐damped state features dominant leakage and stronger resonance coupling, leading to sharper yet more efficient absorption peaks. The influence of other geometric parameters, such as cavity side length on the eigenvalue evolution is discussed in the Section S6. This eigenvalue evolution provides a clear mathematical framework for classifying the damping state of the unit, forming the basis for analyzing its temperature‐dependent transition in the subsequent figures.

Following the identification of the damping conditions in Figure 3A, the temperature‐dependent absorption behavior of the over‐damped and under‐damped TPAM is further analyzed. For the over‐damped configuration shown in Figure 3B, the absorption peak shifts toward higher frequencies under left incidence as the temperature increases from 293 to 493 K. However, the overall absorption level decreases and the peak becomes flatter, indicating that enhanced thermoviscous losses suppress the resonant amplitude despite the moderate broadening of bandwidth. Despite these thermal variations, the right‐incidence response maintains nearly zero absorption, and the overall absorption level remains stable, confirming the robustness of the loss‐asymmetric design. For the under‐damped configuration shown in Figure 3C, the resonance frequency also shifts upward, accompanied by a noticeable increase in bandwidth. Importantly, the absorption peak increases with temperature, as the balance between leakage and intrinsic loss moves closer to the critical coupling condition. This contrasting trend between the two damping states highlights that temperature variations can either suppress or promote absorption by modulating the competition between intrinsic loss and external leakage, which in turn determines whether the system approaches or departs from the critical coupling condition. The consistent agreement between theory and simulation confirms the validity of the temperature‐dependent acoustic model.

To quantitatively compare the temperature effects, Figure 3D presents the variations in maximum absorption peak value and wavelength‐to‐thickness ratio (λ/thickness) for the over‐damped and under‐damped configurations. As the temperature increases from 293 to 493 K, the peak value of the over‐damped unit decreases significantly, whereas that of the under‐damped unit shows a significant increase, reflecting their opposite responses to thermally induced damping variation. Moreover, even with a 200 K increase in temperature, both damping states exhibit high absorption efficiency, with peak values remaining above 80%. The over‐damped configuration consistently exhibits a larger wavelength‐to‐thickness ratio than the under‐damped one under both thermal conditions. While this ratio decreases with increasing temperature, both configurations remain deep subwavelength absorbers with high efficiency in the low‐frequency regime.

It is worth noting that, as shown in Figure 3E, the total energy dissipation increases with temperature for both damping states, but their absorption behaviors evolve in opposite directions. In the over‐damped unit, the intrinsic loss dominates over the radiative leakage. As the temperature rises, the increased damping strength enhances the total energy dissipation but simultaneously disturbs the loss‐leakage balance, driving the system further away from the critical coupling condition. Consequently, the absorption peak decreases despite the higher overall dissipation. In contrast, in the under‐damped configuration, the intrinsic loss at lower temperatures is initially insufficient to balance the external radiation leakage. Thermal elevation enhances the viscous and thermal damping, bringing the loss and leakage into closer balance and driving the system toward the critical coupling condition. As a result, both the dissipation and the absorption peak increase simultaneously. This contrasting behavior shows that absorption depends not on the total energy dissipation but on the balance between loss and leakage. And the respective contributions of viscous and thermal dissipation under different temperatures and damping states are further quantified and discussed in Section S7. Temperature, as an effective non‐geometric parameter, modulates this balance and enables continuous tuning of the absorption response between the over‐ and under‐damped states. To further illustrate the practical implications of this mechanism, corresponding analysis demonstrating fixed‐amplitude frequency modulation and fixed‐frequency amplitude modulation under different temperature conditions are provided in the Section S8, Supplementary Information.

To visualize the underlying mechanisms of the temperature‐dependent response, Figure 3F,G present the model geometry and the corresponding sound‐pressure distributions for the two damping states. As illustrated in Figure 3F, a periodic acoustic unit containing perforated plates is excited by left‐side incidence, and the pressure field is sampled on the central longitudinal plane parallel to the propagation direction. The modulation parameters are temperature *T* and pore diameter *d*, while other geometric dimensions remain fixed. The sound pressure field distributions in Figure 3G, simulated using the same geometric parameters as those adopted in Figure 3B,C, clearly corroborate the corresponding spectral trends. For the under‐damped configuration, the temperature increase from 293 to 493 K causes the resonance frequency to rise (from 400 to 510 Hz) and significantly strengthens the pressure difference amplitude inside the pores, as evidenced by the higher‐pressure contrast between the inlet and the resonator chamber. This trend is fully consistent with the rise of the dissipation peak and the enhanced absorption observed in Figure 3C, confirming that the system approaches the critical‐coupling condition at elevated temperature. For the over‐damped configuration, the resonance shifts from 245 to 310 Hz as temperature increases. Although the overall pressure amplitude rises markedly, the pressure contrast across the neck becomes stronger at elevated temperature, indicating enhanced local confinement of sound energy near the entrance. However, the pressure field within the cavity becomes more spatially diffuse, implying that the internal resonance is suppressed and energy is mainly dissipated near the neck region. Such behavior occurs when most of the acoustic energy is confined or dissipated close to the structure–air interface rather than transmitted into the cavity, resulting in a decrease in total absorbed energy despite the increase in local losses [40]. Consequently, the coupling efficiency between the incident wave and the resonant mode deteriorates, leading to the reduced absorption peak observed in Figure 3B.

Overall, the results in Figure 3 demonstrate that temperature governs the interplay between intrinsic loss and external leakage, thereby dictating the effective damping state and absorption efficiency of the loss‐asymmetric TPAM. Although elevated temperature enhances total viscous dissipation, the absorption performance does not always increase correspondingly, as excessive damping can hinder efficient coupling between the incident wave and the cavity resonance. This phenomenon highlights that insufficient energy exchange rather than inadequate dissipation limits the achievable absorption, especially in the over‐damped state. Therefore, to further understand the mechanism behind this non‐monotonic dissipation response, it is necessary to analyze the system from an impedance‐matching perspective, where the resistance and reactance components determine the degree of energy coupling. Building upon this foundation, the next section focuses on the impedance evolution and coupled‐unit design, revealing how thermally tuned resistance‐reactance balance enables broadband and efficient sound absorption across multiple damping states.

### 4.2 Impedance Based Coupling Mechanisms for Thermally Tuned Broadband Absorption

**FIGURE 4:**
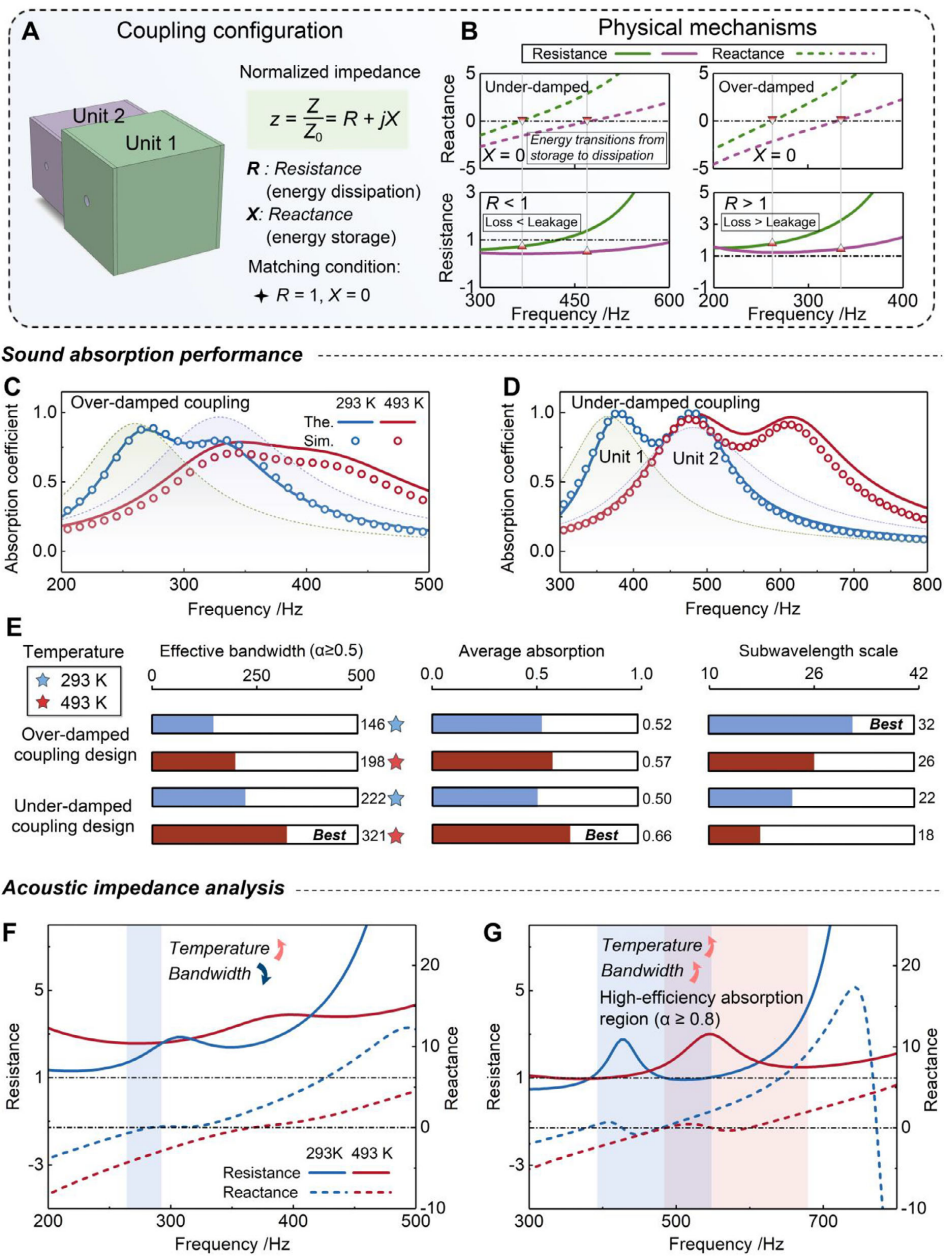
Impedance analysis and thermally programmable coupling mechanisms for broadband sound absorption. (A) Schematic illustration of the coupling configuration, where two acoustic units operating in identical damping states (both over‐damped or both under‐damped) are combined for impedance‐based analysis. (B) Normalized impedance characteristics of representative single units showing the resistance and reactance in the under‐ and over‐damped states. (C,D) Temperature‐dependent absorption spectra of the TPAM under over‐damped and under‐damped coupling configurations. The solid lines represent theoretical predictions and the symbols denote numerical simulations, while the dashed curves correspond to the single‐unit responses. (E) Comparison of three key performance indicators, including effective bandwidth, average absorption, and subwavelength ratio (λ/thickness) between the two coupling designs at 293 and 493 K. (F,G) Acoustic impedance analysis for over‐damped and under‐damped coupling configurations of the TPAM at different temperatures.

As illustrated in Figure 4A, two acoustic units are combined, each designed to operate under the same damping condition: either both in the over‐damped state or both in the under‐damped state, to investigate the impedance characteristics and coupling behavior of the system. The absorbers are characterized by the normalized impedance:

(4) $$z\;=\frac{Z}{Z_{0}}=R+jX$$

where *R* and *X* represent the resistive (dissipative) and reactive (energy‐storage) components, respectively.

This formulation provides an intuitive framework for understanding the temperature‐dependent damping transitions discussed in Section 4.1, linking the balance between intrinsic loss and external leakage to observable impedance behavior. The corresponding impedance characteristics for two single units with different geometrical dimensions are shown in Figure 4B, representing the over‐damped and under‐damped regimes. In this framework, the real part (*R*) quantifies the resistive loss associated with viscous and thermal dissipation, whereas the imaginary part (*X*) represents the reactive component related to energy storage within the resonant cavity [41, 42, 43]. At the condition *X*  =  0, the reactance vanishes, indicating that the stored and radiated acoustic energies are balanced and that the system undergoes a transition from energy storage to energy dissipation. Under this condition, *R* reflects the relative strength of intrinsic loss to external leakage. For the under‐damped state, *R* < 1, indicating that intrinsic losses are weaker than radiation leakage; the system therefore remains leakage‐dominated, and efficient energy exchange occurs near resonance. Conversely, for the over‐damped state, *R* > 1, meaning that intrinsic losses exceed radiation leakage; most of the input acoustic energy is dissipated locally rather than radiated, leading to incomplete energy conversion and reduced absorption efficiency. Maximum absorption is achieved when the impedance becomes purely resistive (*R*  =  1 and *X*  =  0), corresponding to the critical coupling condition that enables maximal acoustic energy transfer into the structure.

To verify the impedance‐based coupling mechanism and the influence of thermal modulation, Figure 4C–E compare the absorption performance of the coupled system under varying temperatures for left‐side incidence. The corresponding absorption spectra and impedance characteristics for right‐side incidence are provided in Section S9. The simulated results are shown as symbols, whereas the solid lines correspond to the theoretical coupled responses of the composite system at 293 (blue) and 493 K (red). The dashed curves indicate the absorption spectra of the two individual units acting independently at 293 K. At the temperature of 293 K, both configurations exhibit distinct absorption peaks originating from the impedance characteristics of each unit, which merge into a single broadband response once the units are coupled. For the over‐damped coupling case (as shown in Figure 4C), increasing temperature shifts the absorption band toward higher frequencies and smooths the spectral profile. The effective bandwidth (α ≥ 0.5) expands from 146 to 198 Hz, and the average absorption improves slightly from 0.52 to 0.57, demonstrating enhanced dissipation. However, the overall absorption remains relatively moderate because excessive viscous loss weakens resonance coupling and suppresses the formation of sharp peaks. In contrast, the under‐damped coupling case (as shown in Figure 4D) shows a markedly different trend. Elevated temperature substantially strengthens the interaction between the two resonant units, causing their peaks to merge and yielding a continuous and wide absorption band. The effective bandwidth expands from 222 to 321 Hz, and the average absorption increases from 0.50 to 0.66, achieving the best overall performance among all conditions. This enhancement arises from improved impedance matching near the critical‐coupling state, where intrinsic loss and radiation leakage are optimally balanced. Figure 4E quantitatively compares three performance indicators: effective bandwidth, average absorption, and subwavelength scale under the two damping regimes and temperatures. The results highlight a clear trade‐off: the under‐damped design at 493 K achieves the broadest and strongest absorption band (Bandwidth =  321, α_*ave*_ =  0.66), while the over‐damped design maintains the most compact subwavelength ratio (λ/thickness =  32). These observations confirm that temperature serves as an efficient external control parameter that adjusts the loss‐leakage balance of each resonant unit, enabling thermally programmable and broadband acoustic absorption through controlled damping‐state coupling. The corresponding geometric parameters are listed in Section S4.

Figure 4F,G provide a detailed analysis of the acoustic impedance to clarify how temperature influences the damping state and absorption bandwidth of the TPAMs. In the over‐damped coupling design (Figure 4F), both the *R* and *X* vary markedly with temperature. At 293 K, the impedance curve exhibits a narrow high‐efficiency absorption region ((α ≥ 0.8, shaded region in blue), where *R* ≈ 1 and *X* ≈ 0, corresponding to near‐critical coupling. When the temperature rises to 493 K, *R* increases far above 1, while *X* approaches zero, indicating that intrinsic viscous losses become dominant and reactive energy storage is largely suppressed. As a result, the high‐efficiency absorption region completely vanishes, confirming that excessive internal dissipation breaks the balance between loss and leakage, thereby eliminating the impedance‐matching condition and reducing overall absorption efficiency despite the slightly broadened frequency response. In contrast, the under‐damped coupling system (as shown in Figure 4G) shows a distinct impedance evolution. As temperature increases, *R* and *X* fluctuate around the matching condition over a much wider frequency range. This corresponds to a significant expansion of the high‐efficiency absorption region at 493 K (α ≥ 0.8, shaded region in red), indicating that elevated temperature effectively balances internal loss and external leakage, thus achieving near‐perfect impedance matching. The resulting broadband and high‐efficiency absorption arises from this temperature‐induced alignment between loss and leakage, which promotes strong and continuous energy dissipation across adjacent resonant modes.

In summary, the impedance analysis reveals that thermal modulation governs the transition between loss‐dominated and leakage‐dominated regimes by continuously tuning the effective resistance and reactance. The over‐damped system benefits from enhanced robustness but limited absorption efficiency at high temperature, whereas the under‐damped system achieves both wider bandwidth and higher absorption due to improved impedance matching. This thermally programmable impedance provides the physical foundation for the broadband absorption demonstrated in this work and establishes a general design principle for achieving high‐temperature sound‐absorbing metastructures. It is worth noting that the coupled configuration analyzed here represents a minimal demonstrative system composed of two resonant units, selected to clearly elucidate the temperature‐regulated loss‐leakage coupling mechanism. Owing to the resonance‐based nature of the two‐port unit, the same impedance‐matching mechanism can be straightforwardly extended to multi‐order coupled architectures [44, 45], thereby enabling broader absorption bandwidths while preserving the underlying physical framework.

### 4.3 Functional Asymmetry Induced by Temperature Differences under Geometrical Symmetry

**FIGURE 5:**
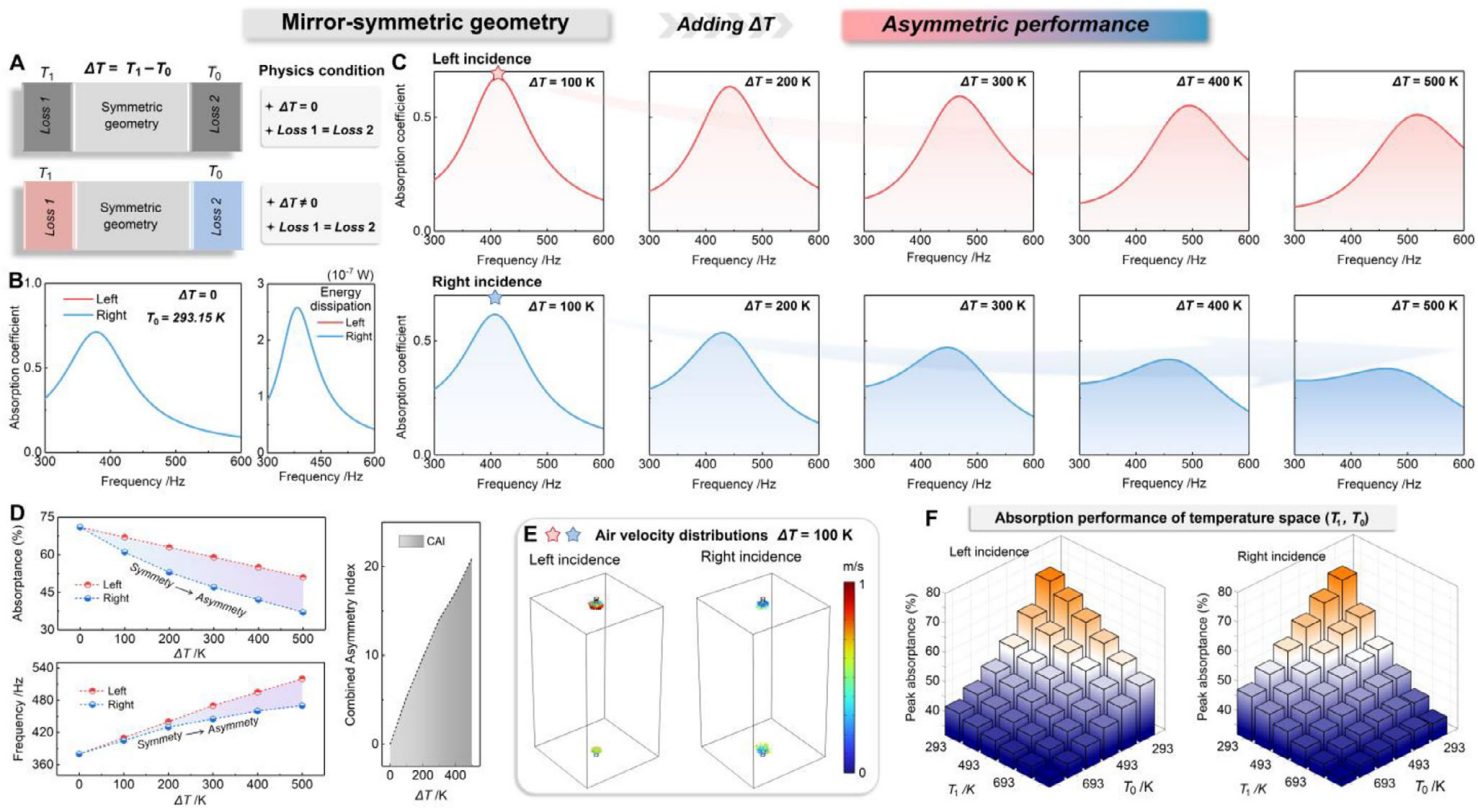
Functional asymmetry induced by temperature differences under geometrical symmetry. (A) Schematic illustration of a mirror‐symmetric two‐port acoustic system under uniform (Δ*T*  =  0 *K*) and non‐uniform (ΔT≠0K) temperature conditions. (B) Baseline case at Δ*T*  =  0 *K*, *T*_1_ = *T*_0_  =  293 *K*, where simulated absorption spectra and energy‐dissipation curves for left and right incidences coincide, confirming fully symmetric acoustic performance. (C) Evolution of simulated absorption spectra as Δ*T* increases from 100 to 500 K, showing that symmetric geometry yields asymmetric absorption responses. (D) Quantitative comparison of peak absorptance and resonance frequency under different Δ*T*, along with the CAI that captures the growth of functional asymmetry with increasing temperature difference. (E) Simulated acoustic particle velocity fields at Δ*T*  =  100 *K*, visualizing stronger energy localization near the hot side under left incidence and weaker dissipation for right incidence. (F) Mapping of peak absorption over 2D temperature space (*T*_1_, *T*_0_), revealing that different temperature combinations jointly determine the asymmetric performance of the two‐port acoustic system.

In the preceding analysis, temperature was introduced as a cooperative parameter influencing the coupling behavior and absorption performance of the two‐port acoustic system. Here, it is further treated as an independent non‐geometric variable for regulating system responses. Temperature has been shown to play a crucial role in governing the coupling strength and absorption performance of two‐port acoustic systems. Beyond purely geometric tuning, thermal modulation offers an additional and versatile degree of freedom to manipulate system losses and wave interactions. Based on this understanding, we further investigate how a temperature gradient, applied without altering the geometry, can give rise to pronounced functional asymmetry. Specifically, even for a structure that is perfectly mirror‐symmetric in shape and boundary configuration, introducing a temperature difference between its two sides inevitably alters the local thermoviscous parameters of air, thereby inducing asymmetric intrinsic losses. This thermal difference induces asymmetric energy dissipation while maintaining geometric symmetry, thereby establishing a non‐geometric pathway toward temperature‐driven functional asymmetry in a passive two‐port non‐Hermitian acoustic system. Figure 5 illustrates this evolution process, showing how a symmetric configuration progressively develops direction‐dependent acoustic responses with the temperature difference Δ*T*.

Figure 5A schematically illustrates the physical conditions of the mirror‐symmetric system under uniform and non‐uniform temperature fields. When Δ*T*  =  0 *K*, both sides are maintained at the same temperature (*T*_1_ = *T*_0_ ), and the thermoviscous parameters of the air medium remain identical. Consequently, the two lossy regions, denoted as Loss 1 and Loss 2, possess equal damping properties, i.e., Loss 1 = Loss 2, ensuring fully symmetric energy dissipation across the structure. Once a temperature gradient is introduced (ΔT≠0K), the geometry and boundary configurations remain unchanged, but the temperature dependence of air parameters causes the effective loss coefficients to deviate (Loss 1 ≠ Loss 2). Although the geometry remains symmetric, the thermally induced imbalance gives rise to markedly asymmetric dissipation responses, revealing the emergence of functional asymmetry in such a two‐port acoustic system. Figure 5B presents the case under uniform temperature conditions (Δ*T*  =  0 *K*, *T*_1_ = *T*_0_  =  293 *K*), where both sides of the system share identical parameters. The geometric parameters are listed in Section S4. Owing to the geometric and physical symmetry, the simulated absorption spectra for left and right incidences perfectly overlap. The corresponding energy‐dissipation curves further demonstrate that the dissipated power is equal for both directions, verifying that the loss distribution remains fully balanced. Under this condition, the system functions as an ideal symmetric two‐port resonator with identical impedance environments on both sides, serving as a reference state for subsequent thermally induced asymmetry.

Figure 5C illustrates the evolution of the absorption spectra, obtained from numerical simulations, when the temperature difference Δ*T* is applied between the two sides, increasing from 100 to 500 K, while the right‐side temperature is maintained at *T*_0_ =  293 *K*. Although the geometric configuration remains strictly mirror‐symmetric, the thermal gradient introduces distinct changes in the acoustic response for the two incidence directions. For left incidence, the absorption peak gradually shifts toward higher frequencies with a moderate reduction in amplitude. In contrast, for right incidence, the absorption peak weakens more markedly with increasing Δ*T*, and the absorption band becomes broader. Compared with the uniform‐temperature case in Figure 5B, these results highlight that a temperature gradient alone is sufficient to induce directional differences in absorption performance, even without any change in geometry. Further, Figure 5D provides a quantitative assessment of the asymmetric absorption behaviors. The upper panel plots the variation of peak absorptance for left and right incidences as a function of the Δ*T*. As Δ*T* increases, the absorptance associated with left incidence shows a gradual enhancement, whereas that for right incidence monotonically decreases, confirming the opposite evolution tendencies induced by the temperature gradient. The lower panel presents the corresponding resonance frequencies extracted from both incidence cases. The resonant frequencies of both directions shift toward higher values as the temperature difference increases, owing to the temperature‐dependent variation in sound speed and cavity impedance. However, a clear frequency divergence is observed: the shift is more pronounced for left incidence, indicating a faster modulation of the resonant condition on the hot side. This asymmetric rate of frequency variation reveals that the temperature gradient not only affects the magnitude of absorption but also induces direction‐dependent tuning of the resonant response. To quantitatively integrate both amplitude and frequency disparities, a Combined Asymmetry Index (CAI) is introduced and defined as:

(5) $$CAI=\sqrt{\frac{1}{2}\left[{\left(\frac{\alpha_{L}-\alpha_{R}}{\alpha_{L}+\alpha_{R}}\right)}^{2}+{\left(\frac{f_{L}-f_{R}}{f_{L}+f_{R}}\right)}^{2}\right]}\;$$

The CAI grows monotonically with Δ*T*, as shown in the shaded region of Figure 5D, quantitatively capturing the transition from a symmetric to an asymmetric state. This increase in CAI signifies a progressive enhancement of functional asymmetry, driven solely by the temperature‐induced loss imbalance, and provides a concise metric for evaluating the asymmetric performance of the two‐port acoustic system. To further illustrate this directional difference more clearly, Figure 5E takes the case of Δ*T*  =  100 *K* as an example, showing the acoustic particle velocity fields for left and right incidences. For left incidence, the velocity field exhibits strong localization near the hot‐side cavity, where elevated air viscosity enhances viscous and thermal dissipation. This leads to higher velocity amplitudes and concentrated energy circulation within the near‐wall region. In contrast, for right incidence, the velocity distribution becomes more diffuse with lower local amplitudes, indicating weaker coupling and reduced energy confinement near the cold side. These contrasting field patterns clearly demonstrate that the temperature gradient induces direction‐dependent energy flow and dissipation intensity, offering an asymmetric absorption behavior in the geometrically symmetric structure.

Figure 5F extends the analysis by mapping the absorption performance over a 2D temperature space, where the left‐side temperature (*T*_1_) and right‐side temperature (*T*_0_) are varied independently. While Figure 5C focuses on the representative case of a hot‐left and cold‐right configuration, Figure 5F generalizes this condition to examine all possible temperature combinations. The color surface represents the simulated peak absorption coefficient under each pair of (*T*_1_,*T*_0_) values. As both temperatures increase, the overall absorption level gradually rises due to the enhanced viscous losses of air at elevated temperatures. More importantly, the two incidence directions display distinct surface morphologies, revealing that the degree of asymmetry depends jointly on the absolute temperatures and their difference. This mapping provides a comprehensive view of how thermal parameters jointly govern the absorption characteristics in the two‐port open acoustic system, enabling predictive control of directional performance through temperature modulation.

In summary, this section demonstrates that a temperature gradient can induce clear functional asymmetry in a geometrically symmetric two‐port acoustic system. As the temperature difference increases, the temperature‐dependent loss imbalance causes direction‐dependent variations in absorption magnitude and resonance frequency. The resulting asymmetry, visualized by particle‐velocity fields and quantified by the CAI metric, highlights temperature as an effective non‐geometric parameter for tuning the bidirectional acoustic response.

### 4.4 Scientific Advance

**FIGURE 6:**
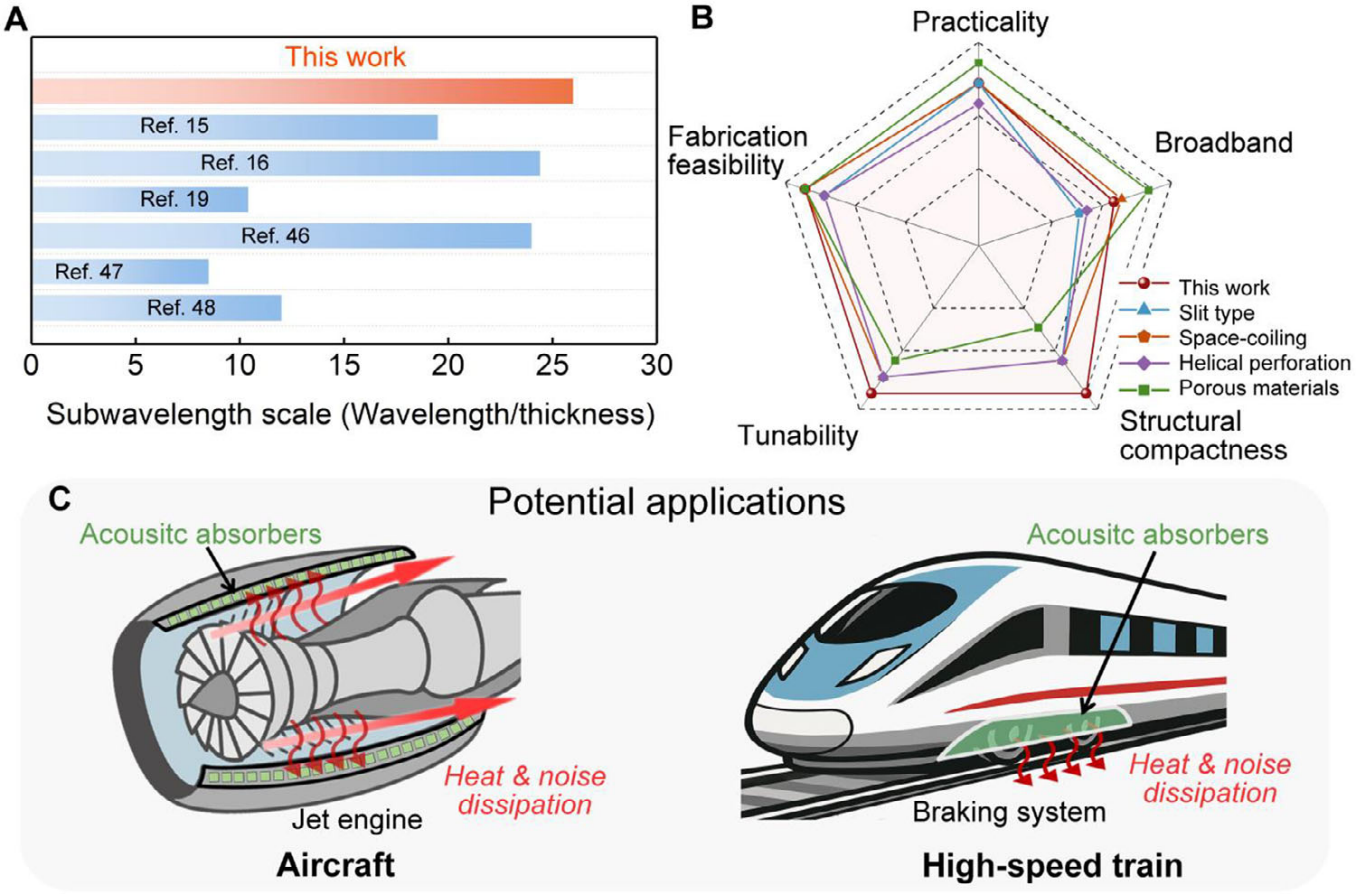
Scientific advances and potential applications of the proposed TPAM. (A) Comparison of subwavelength ratios between this work and representative high‐temperature or thermally robust acoustic absorbers reported in previous studies, with a focus on low‐frequency operating regimes. (B) Radar plot comparison of key evaluation criteria, highlighting the thermally programmable characteristics of the proposed metastructure in comparison with representative high‐temperature acoustic metamaterial absorbers. (C) Potential engineering applications in high‐temperature and noise‐intensive environments, such as aircraft engines and high‐speed train braking systems.

This work establishes a thermal‐acoustic coupling framework that expands the functional limits of conventional geometrically tuned acoustic systems. By integrating structural and thermal degrees of freedom, the proposed TPAM realizes temperature‐driven, direction‐dependent acoustic responses and broadband absorption in a compact two‐port system, by exploiting controllable asymmetry in intrinsic loss within a passive non‐Hermitian framework. Rather than being a detrimental disturbance, the temperature field functions as an active non‐geometric parameter that enables precise control of the damping state and impedance matching of the coupled resonators, thereby inducing asymmetric energy dissipation in geometrically symmetric systems. At elevated temperatures, the enhancement of thermoviscous losses promotes strong and continuous impedance matching, yielding efficient broadband absorption without sacrificing subwavelength compactness. To place this framework in a broader scientific and engineering context, Figure 6 summarizes comparative performance metrics and representative application scenarios, highlighting the advantages of the proposed design over existing acoustic absorbers and its adaptability to extreme thermal environments.

In low‐frequency regimes characterized by long wavelengths and weak intrinsic dissipation in air, compact and thermally robust acoustic absorption remains a central design objective. Under high‐temperature operating conditions, this challenge is commonly addressed through structurally compact designs combined with enhanced thermoviscous dissipation mechanisms. As shown in Figure 6A, the proposed design is presented together with representative high‐temperature‐resistant or thermally robust acoustic absorbers reported in previous studies [15, 16, 19, 46, 47, 48], to verify that the thermally programmable metastructure exhibits a comparable subwavelength scale under elevated‐temperature conditions around 493 K. It is observed that the proposed structure maintains a deeply subwavelength thickness of approximately λ/26 in the low‐frequency regime, which is comparable to the characteristic scale of existing high‐temperature acoustic absorbers, confirming its practical compactness under elevated‐temperature operation.

Figure 6B further provides a qualitative positioning of the proposed design among representative high‐temperature acoustic absorbers using a radar plot representation [15, 16, 46, 48]. Instead of ranking performance, this comparison aims to illustrate the overall balance of design attributes of the proposed metastructure, including absorption bandwidth, structural compactness, tunability, fabrication feasibility, and applicability under elevated‐temperature environments. The radar‐plot visualization indicates that, through the combined regulation of geometric configuration and temperature‐dependent loss modulation, the proposed design maintains a well‐coordinated performance profile across these key metrics. Corresponding quantitative data used for the radar plot construction are summarized in Section S10.

Building on these performance comparisons, the temperature‐adaptive mechanism of the proposed absorber opens promising opportunities for real‐world applications [49, 50, 51, 52, 53]. It should be emphasized that temperature is not intentionally adjusted or locally controlled in this work, but instead reflects thermal conditions that naturally arise during operation in many high‐temperature scenarios, so that the acoustic response adapts passively without additional heating, cooling, or thermal‐management systems. In environments where high temperature and strong noise coexist, such as aerospace propulsion systems and high‐speed railway braking systems, these naturally occurring thermal conditions allow the structure to maintain efficient absorption by exploiting temperature‐enhanced viscous dissipation. From an experimental implementation perspective, although the present experimental validation is conducted at room temperature, the proposed thermally programmable non‐Hermitian framework is not intrinsically limited to ambient conditions. High‐temperature acoustic measurements have been demonstrated in recent studies using modified waveguide‐based or planar configurations, in which global heating of the specimen‐air system is realized while thermally isolating the sound source and sensing electronics [54]. Within such established experimental paradigms, temperature‐dependent acoustic responses can be reliably characterized through controlled heating, thermal insulation, and in situ temperature calibration. This provides a clear and feasible pathway for extending the present design toward elevated‐temperature experimental validation in future work.

Although several existing acoustic metastructures have demonstrated excellent absorption performance in similar application scenarios, their designs generally neglect the influence of elevated temperatures and do not incorporate the thermal environment as an active design factor. In contrast, the proposed approach leverages thermally enhanced viscous dissipation to sustain and even improve acoustic efficiency under high‐temperature conditions. This intrinsic adaptability not only ensures stable operation in extreme thermal‐acoustic environments, but also exemplifies a non‐geometric design paradigm for multifunctional thermal‐acoustic management, bridging fundamental physical mechanisms with practical engineering demands.

### 4.5 Conclusions

In summary, a thermally programmable two‐port non‐Hermitian acoustic metastructure has been theoretically and numerically investigated. By introducing temperature as an independent non‐geometric parameter, this study demonstrates that thermal modulation can effectively regulate the damping state and adjust the balance between intrinsic loss and external leakage, enabling broadband absorption within a deep subwavelength thickness. The results reveal that temperature variation provides a controllable means to tailor impedance matching and directional acoustic response, transforming heat from an environmental disturbance into a design variable. Moreover, the emergence of functional asymmetry under geometrical symmetry highlights a non‐geometric mechanism for achieving temperature‐dependent and direction‐selective absorption. Notably, this temperature‐driven direction dependence does not rely on structural asymmetry, but originates from asymmetric loss modulation induced by thermal variation. This feature provides additional flexibility for directional sound control in thermally heterogeneous environments. These findings collectively validate the feasibility of using thermal environments as an additional design dimension for compact and adaptive sound‐absorbing composites.

The present study is primarily aimed at revealing the underlying thermo‐acoustic mechanism rather than developing a specific high‐temperature material system. The prototype fabricated using polylactic acid (PLA) serves to validate the proposed loss‐leakage coupling framework under well‐controlled room‐temperature conditions. Since the acoustic response is governed by air‐structure interactions and thermoviscous dissipation, while the solid frame can be treated as acoustically rigid, the proposed mechanism is not intrinsically tied to the material choice. For practical high‐temperature applications, the same design can be implemented using thermally stable materials such as high‐temperature resins, metallic alloys, or ceramic‐based composites, without altering the fundamental absorption mechanism. Although high‐temperature experiments are not conducted in the present work, the strong agreement between theory, simulation, and experiment supports the validity of the proposed framework. Extending the present design toward elevated‐temperature experimental validation therefore constitutes a natural and feasible direction for future studies.

### 4.6 Design and Fabrication

The acoustic metastructures were fabricated from PLA using a commercial fused deposition modeling (FDM) 3D printer (Bambu Lab A1 Series). The CAD geometries were first created in SolidWorks and exported as STL files for additive manufacturing. Printing was performed with a 0.4 mm brass nozzle at a temperature of 488 K and a platform temperature of 333 K. A layer height of 0.1 mm was selected to ensure geometric precision and structural integrity. The printed samples exhibited high dimensional accuracy with negligible deviation from the designed geometry, providing reliable prototypes for subsequent acoustic characterization.

### 4.7 Numerical Simulations

Numerical analyses were performed in COMSOL Multiphysics by coupling the Pressure Acoustics and Thermoviscous Acoustics modules to capture both wave propagation and dissipative effects within the microperforated structure. As described in Section S1, the background medium was defined as air, whose thermophysical properties including density, sound speed, and dynamic viscosity were specified as temperature‐dependent variables. The Pressure Acoustics module governs the propagation of sound waves, while the Thermoviscous Acoustics module accounts for thermal conduction and viscous losses occurring inside sub‐millimeter perforations. The reflection, transmission, and absorption coefficients were calculated using the TMM. The transmission coefficient was obtained from the ratio of transmitted to incident sound power across the two‐port boundaries, and the absorption and reflection coefficients were subsequently derived as α  =  1 − |*r*|^2^ − |*t*|^2^.

### 4.8 Experiments

The absorption coefficients were measured in a custom‐built rectangular impedance tube (145 mm × 145 mm) following ASTM E2611–17 [31]. White noise was generated by a loudspeaker and amplified with a B&K Type 2734‐A amplifier. Sound pressures were recorded using 1/4‐inch B&K Type 4494‐A microphones connected to a B&K LAN‐XI Light data acquisition module (Type 3677). The valid frequency range, calculated from microphone spacing (50 mm) and tube size (145 mm), was 68.6 Hz < *f* < 1182.8 Hz. Absorption coefficients were obtained via the standard transfer‐function method.

## Conflicts of Interest

The authors declare no conflicts of interest.

## Supporting information




**Supporting File**: advs74047‐sup‐0001‐SuppMat.docx.

## Data Availability

The data that support the findings of this study are available from the corresponding author upon reasonable request.
