# Supplementary material for: Thermally Programmable Two‐Port Non‐Hermitian Acoustic Metastructure for Broadband and Direction‐Dependent Absorption
Source: Adv Sci (Weinh). 2026 Jan 28;13(19):e24228. doi: 10.1002/advs.202524228 (PMC13045492; doi:10.1002/advs.202524228)
Supplement: Supplementary file 1 — Supporting File: advs74047‐sup‐0001‐SuppMat.docx. [file ADVS-13-e24228-s001.docx]

**Supporting Information**

**Thermally Programmable Two-Port Non-Hermitian Acoustic Metastructure for Broadband and Direction-Dependent Absorption**

Zichao Guo, Zhendong Li*, Ziping Lei, Kexin Zeng, Zheng Fan*, Zhonggang Wang *

## Section S1. Derivation of Temperature-Dependent Parameters in the Analytical Model

The temperature-dependent parameters of air used in the analytical model are calculated as follows. The sound speed $c_{0}$ is given by

|  | $c_{0}=\sqrt{\gamma\frac{R}{M}T}$ | (S1) |
| --- | --- | --- |

where $\gamma$ is the specific heat ratio, $R$ is the universal gas constant, $M$ is the molar mass of air, and $T$ is the absolute temperature.

The equilibrium air density $\rho_{0}$ is expressed as

|  | $\rho_{0}=\frac{P_{0}M}{RT}$ | (S2) |
| --- | --- | --- |

where $P_{0}$is the ambient pressure.

The dynamic viscosity $\mu$ of air varies with temperature according to Sutherland’s formula [S1]:

|  | $\mu=\eta_{ref}\frac{T_{ref}+C}{T+C}\left( \frac{T}{T_{ref}} \right)^{3/2}$ | (S3) |
| --- | --- | --- |

where $\eta_{ref}$ and $T_{ref}$ are the reference viscosity and temperature, respectively, and $C$ is Sutherland’s constant.

Finally, the characteristic acoustic impedance $Z_{0}(T)$ is determined as

|  | $Z_{0}(T)=\rho_{0}c_{0}$ | (S4) |
| --- | --- | --- |

These relations collectively describe how temperature influences the basic thermophysical properties of air, including density, viscosity, and acoustic impedance, which are incorporated into the analytical and numerical models throughout this work.

## Section S2. Detailed derivations of the analytical model

We employ the parallel transfer‐matrix method (TMM) to calculate the acoustic response of asymmetric absorbers [S2], where temperature‐dependent air parameters are incorporated to account for thermal modulation effects

|  | $M_{Parallel}^{t}$=$\left[ \begin{matrix} {A\left( T,f \right)}^{t} & {B\left( T,f \right)}^{t} \\ {C\left( T,f \right)}^{t} & {D\left( T,f \right)}^{t} \end{matrix} \right]=\frac{-1}{\sum\delta_{i}Y_{21}^{i}}\left( \begin{matrix} \sum\delta_{i}Y_{21}^{i} & -1 \\ \sum\delta_{i}Y_{22}^{i}\sum\delta_{i}Y_{11}^{i}-\sum\delta_{i}Y_{12}^{i}\sum\delta_{i}Y_{21}^{i} & -\sum\delta_{i}Y_{11}^{i} \end{matrix} \right)$ | (S5) |
| --- | --- | --- |

|  | $Y^{i}=\left[ \begin{matrix} Y_{11}^{i} & Y_{12}^{i} \\ Y_{21}^{i} & Y_{22}^{i} \end{matrix} \right]=\frac{1}{B\left( T,f \right)}\left[ \begin{matrix} D\left( T,f \right) & B\left( T,f \right)C\left( T,f \right)-A\left( T,f \right)D\left( T,f \right) \\ 1 & -A\left( T,f \right) \end{matrix} \right]$ | (S6) |
| --- | --- | --- |

where $Y^{i}$ and $\delta_{i}$ refer to the admittance matrix and area ratio of each asymmetric unit with the parallel absorbers, respectively. The total transfer matrix $M$ of single unit is expressed as

|  | $M=\left[ \begin{matrix} A\left( T,f \right) & B\left( T,f \right) \\ C\left( T,f \right) & D\left( T,f \right) \end{matrix} \right]=M_{mpp}^{L,i}M_{cavity}^{i}M_{mpp}^{R,i}$ | (S7) |
| --- | --- | --- |

where $M_{mpp}^{L,i}$ and $M_{mpp}^{R,i}$ describe the matrices of the plates, respectively; $M_{cavity}^{i}$ represents the matrix of the cavity, calculated as

|  | $M_{mpp}=\left[ \begin{matrix} 1 & Z_{p} \\ 0 & 1 \end{matrix} \right]$ | (S8) |
| --- | --- | --- |
|  | $M_{cavity}=\left[ \begin{matrix} cos(k(T)H) & jZ_{0}(T)sin(k(T)H) \\ j\sin\left( k(T)H \right)/Z_{0}(T) & cos(k(T)H) \end{matrix} \right]$ | (S9) |

$Z_{p}$ denotes the impedance of MPP and is expressed [S3]

|  | $Z_{p}=\frac{j\omega\rho_{0}t}{\sigma_{i}}\left[ 1-\frac{2B_{1}(\eta(T)\sqrt{-j})}{(\eta(T)\sqrt{-j})B_{0}(\eta(T)\sqrt{-j})} \right]^{-1}+\frac{\sqrt{2}\mu\eta(T)}{\sigma_{i}d_{i}}+\frac{0.85j\omega\rho_{0}d_{i}}{\sigma_{i}}$ | (S10) |
| --- | --- | --- |

where $\eta(T)=d_{L/R,i}\sqrt{\omega\rho_{0}/4\mu}$ represents the ratio of the diameter of the perforation to the thickness of the viscous boundary layer. $B_{0}$ and $B_{1}$ are the zeroth and first order Bessel functions, respectively.

The temperature-dependent scattering coefficients of the metastructure are expressed as [S4]

|  | $t=\frac{2e^{jk\left( T \right)L}}{\sum\left( T,f \right)}$ | (S11) |
| --- | --- | --- |
|  | $r_{L}=\frac{A\left( T,f \right)+\frac{B\left( T,f \right)}{Z_{0}\left( T \right)}-C\left( T,f \right)Z_{0}\left( T \right)-D\left( T,f \right)}{\sum\left( T,f \right)}$ | (S12) |
|  | $r_{R}=\frac{-A\left( T,f \right)+\frac{B\left( T,f \right)}{Z_{0}\left( T \right)}-C\left( T,f \right)Z_{0}\left( T \right)+D\left( T,f \right)}{\sum\left( T,f \right)}$ | (S13) |
|  | $\sum(T,f)=A\left( T,f \right)+\frac{B\left( T,f \right)}{Z_{0}\left( T \right)}+C\left( T,f \right)Z_{0}\left( T \right)+D\left( T,f \right)$ | (S14) |

where $r_{L}$and $r_{R}$denote the reflection coefficients for left and right incidence, respectively, and $t$ represents the transmission coefficient. The matrix elements $A(T,f)$, $B(T,f)$, $C(T,f)$, and $D(T,f)$are temperature-dependent transfer-matrix terms that account for the changes in air properties (density, viscosity, and sound speed) as described in Section S1.

The characteristic impedance $Z_{0}(T)$and the wavenumber $k(T)$are also temperature-dependent, ensuring that both the propagation and dissipation behaviors are accurately captured under varying thermal conditions. This formulation establishes a unified analytical framework that directly links temperature variation to the scattering and absorption characteristics of the metastructure.

## Section S3. Sound absorption measurements

The cross-section of the waveguide is 145 mm × 145 mm. A loudspeaker positioned at one end of the tube generated white noise, which was amplified using a Brüel & Kjær (B&K) Type 2734-A power amplifier to produce incident waves in the waveguide. 1/4-inch condenser microphones (B&K Type 4494-A) were used to measure the sound pressure at designated positions according to the requirements of the transfer-function method. The data were acquired using a LAN-XI Light 4-channel data acquisition module (Type 3677).

The sound absorption coefficients were measured in a custom-built rectangular acrylic waveguide using a digital frequency analysis system, following the ASTM E2611-17 standard.. The schematic of the impedance tube is shown in Figure S6. The impedance tube used in this study was custom-built, and therefore the valid measurement frequency range was calculated based on the microphone spacing and tube diameter according to ASTM E2611-17 [S5]:

|  | $f_{l}=\frac{1\%c}{s},f_{u}=\frac{50\%c}{d},$ | (S15) |
| --- | --- | --- |

where $c=343 m/s$ is the speed of sound, $s=50 mm$ is spacing between microphones, and $d=145 mm$ is the side size of tube. This yields a valid measurement frequency range of $68.6 Hz<f<1182.8\mathrm{Hz}$, which is adopted in this work. The normal-incidence absorption coefficient was then obtained following the standard procedure [S6, S7]. All specimen and setup photographs were taken using a Nikon Z6 Ⅱ camera.


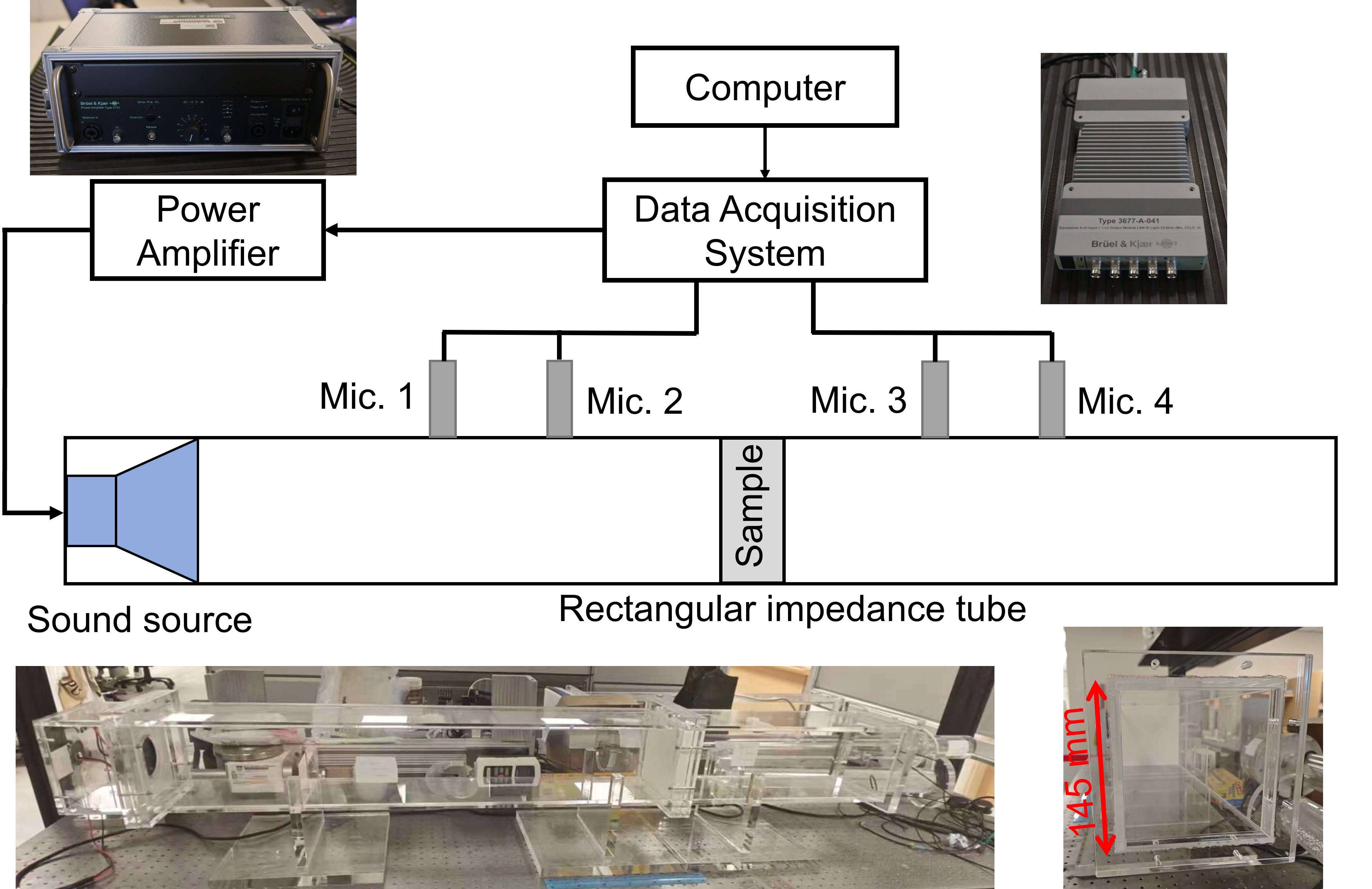


**Figure S1.** Schematics of the impedance waveguide.

## Section S4. Geometric configurations and parameters of proposed design

**Table S1** Geometric parameters of experimental specimen. (Unit: mm)

|  | $d_{L,1}$ | $t_{L}$ | $d_{R,1}$ | $t_{R}$ | $H$ | $L$ | $T$ |
| --- | --- | --- | --- | --- | --- | --- | --- |
| Specimen | $2\times1.0$ | 1 | 1.0 | 1 | 38 | 23 | 293 K |

*Note: The notation “*$2\times1.0$*” indicates that two pores of 1.0 mm are symmetrically arranged.*

**Table. S2** Geometric parameter of proposed acoustic metastructure in Fig. 2. (unit: mm).

|  | $d_{L,1}$ | $t_{L}$ | $d_{R,1}$ | $t_{R}$ | $H$ | $L$ | $T$ |
| --- | --- | --- | --- | --- | --- | --- | --- |
| Prototype | 1.103 | 1 | 0.253 | 1 | 38 | 20 | 293 K |

**Table S3** Geometric parameters of coupling configuration. (Unit: mm)

|  | $d_{L,1}$ | $d_{L,2}$ | $t_{L}$ | $d_{R,1}$ | $d_{R,2}$ | $t_{R}$ | $H$ | $L_{1}$ | $L_{2}$ |
| --- | --- | --- | --- | --- | --- | --- | --- | --- | --- |
| Over-damped coupling | 1.1 | 1.0 | 1 | 0.6 | 0.6 | 1 | 38 | 40 | 30 |
| Under-damped coupling | 1.4 | 1.35 | 1 | 0.6 | 0.6 | 1 | 38 | 40 | 30 |

**Table. S4** Geometric parameter of proposed acoustic metastructure in Fig. 5. (unit: mm).

| $d_{L,1}$ | $t_{L}$ | $d_{R,1}$ | $t_{R}$ | $H$ | $L$ |
| --- | --- | --- | --- | --- | --- |
| 1.0 | 1 | 1.0 | 1 | 38 | 20 |

## Section S5. Evolution of the real and imaginary parts of the eigenvalues ($\boldsymbol{\lambda}_{\boldsymbol{\pm}}$)


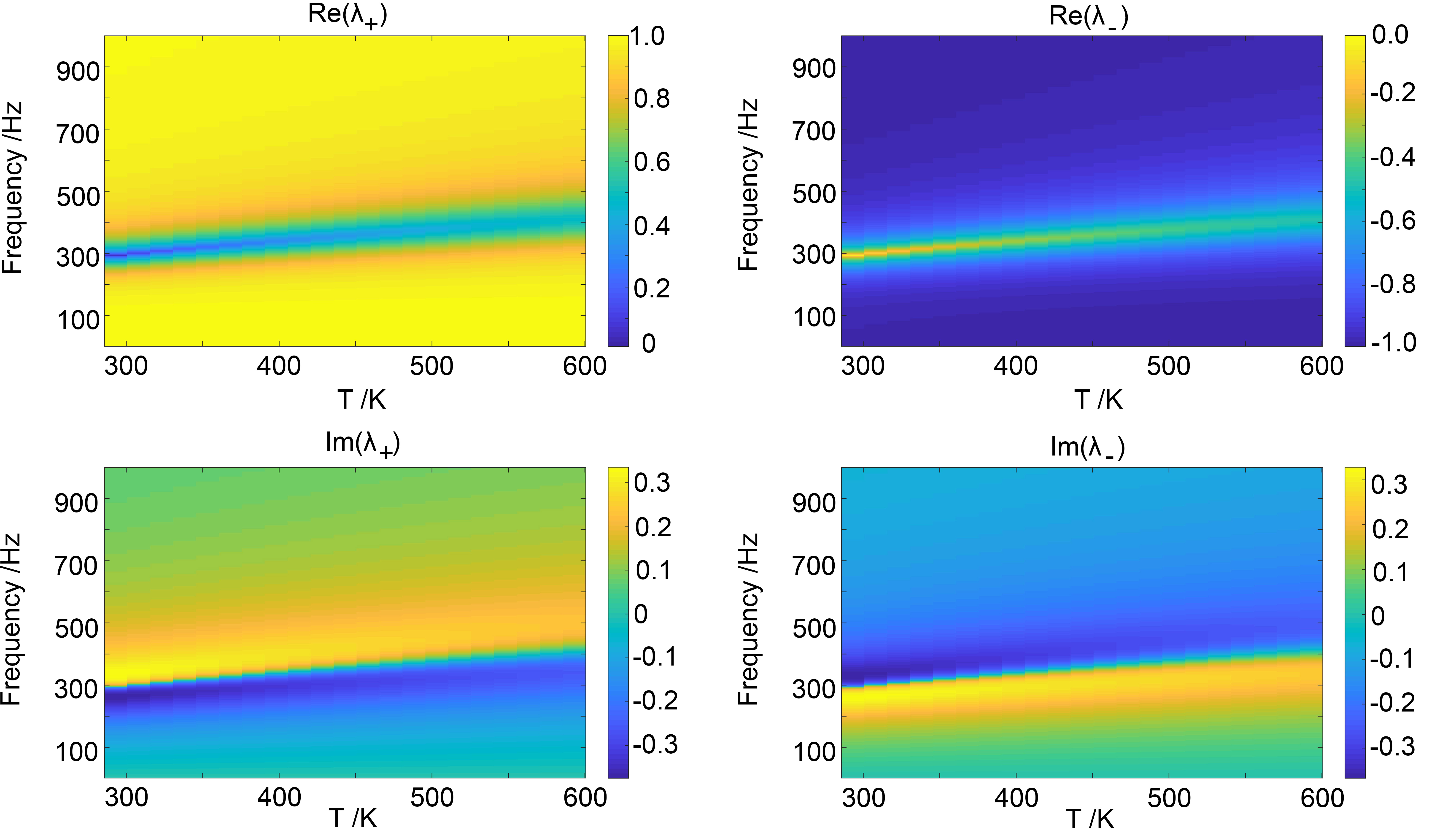


**Figure S2.** Temperature-dependent evolution of the real and imaginary parts of the eigenvalues ($\lambda_{\pm}$) as functions of frequency and temperature, showing thermally induced shifts in resonance frequency and modal loss that characterize non-Hermitian coupling in the loss-asymmetric metastructure.

## Section S6. Eigenvalue evolution as a function of cavity length


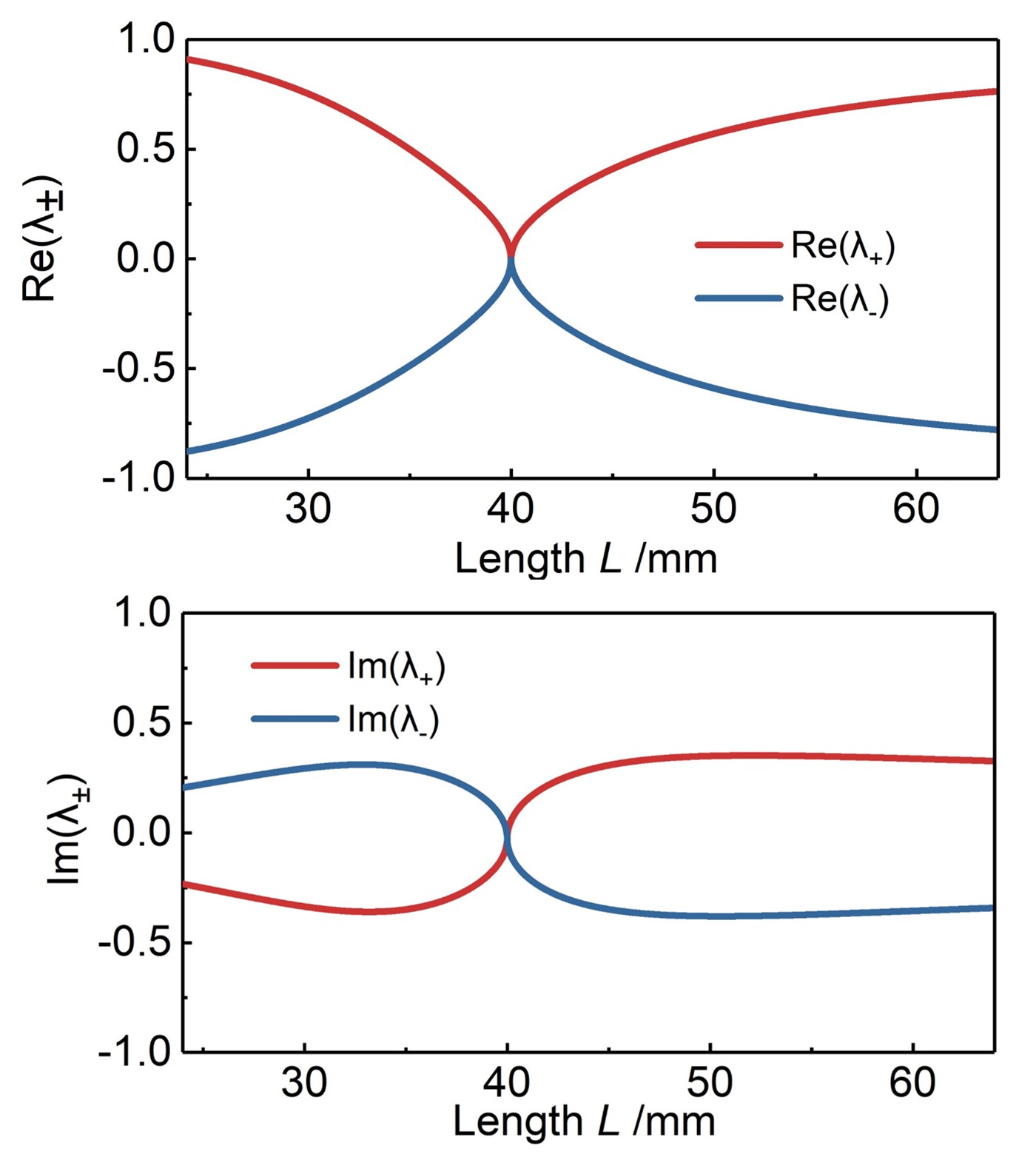


**Figure S3.** Eigenvalue evolution as a function of cavity length (*L*) at room temperature.

The real and imaginary parts of the eigenvalues are plotted against the cavity length *L* to illustrate the influence of structural dimensions on the coupling state. As *L* increases, the system transitions from an under-coupled to a critically coupled regime, where the two eigenvalue branches coalesce at an exceptional point. This result confirms that geometric parameters such as cavity length play a key role in determining the location of the EP and the balance between loss and leakage in the metastructure.

## Section S7. Thermal and viscous dissipation analysis under different damping states and temperatures.

The total thermoviscous energy dissipation is decomposed into thermal dissipation and viscous dissipation components to clarify their respective roles under different damping regimes and temperatures. Thermal dissipation originates from irreversible thermal flux within the thermal boundary layers, reflecting the breakdown of adiabatic sound propagation in confined cavities. In contrast, viscous dissipation arises from frictional motion and strong velocity gradients of oscillatory airflow near the micro-perforations, where shear effects are maximized at resonance.

**Figure S4** presents the frequency-dependent thermal and viscous dissipation spectra for both over-damped and under-damped states at 293 K and 493 K. For all considered conditions, the total energy dissipation is predominantly governed by viscous dissipation, whereas thermal dissipation contributes comparatively weakly. With increasing temperature, both dissipation channels are enhanced due to temperature-dependent air properties; however, viscous dissipation remains the dominant loss mechanism across the entire frequency range. These results further confirm that sound absorption in the proposed structure is primarily controlled by viscous losses in the perforated channels, while thermal dissipation in the cavities plays a secondary role.


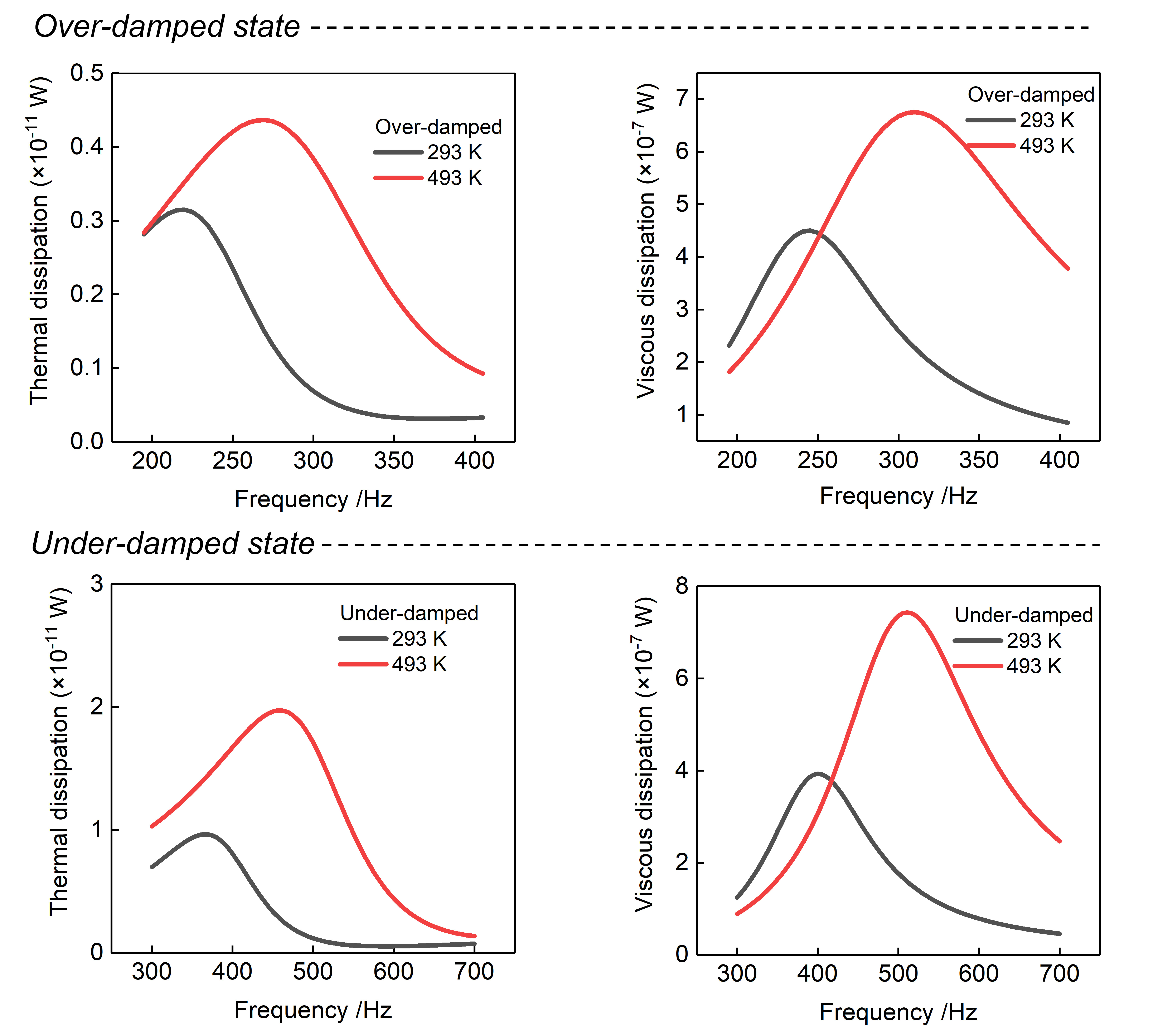


**Figure S4.** Viscous and thermal dissipation under over-damped and under-damped states at different temperatures.

## Section S8 Geometry-assisted temperature regulation of absorption frequency and amplitude


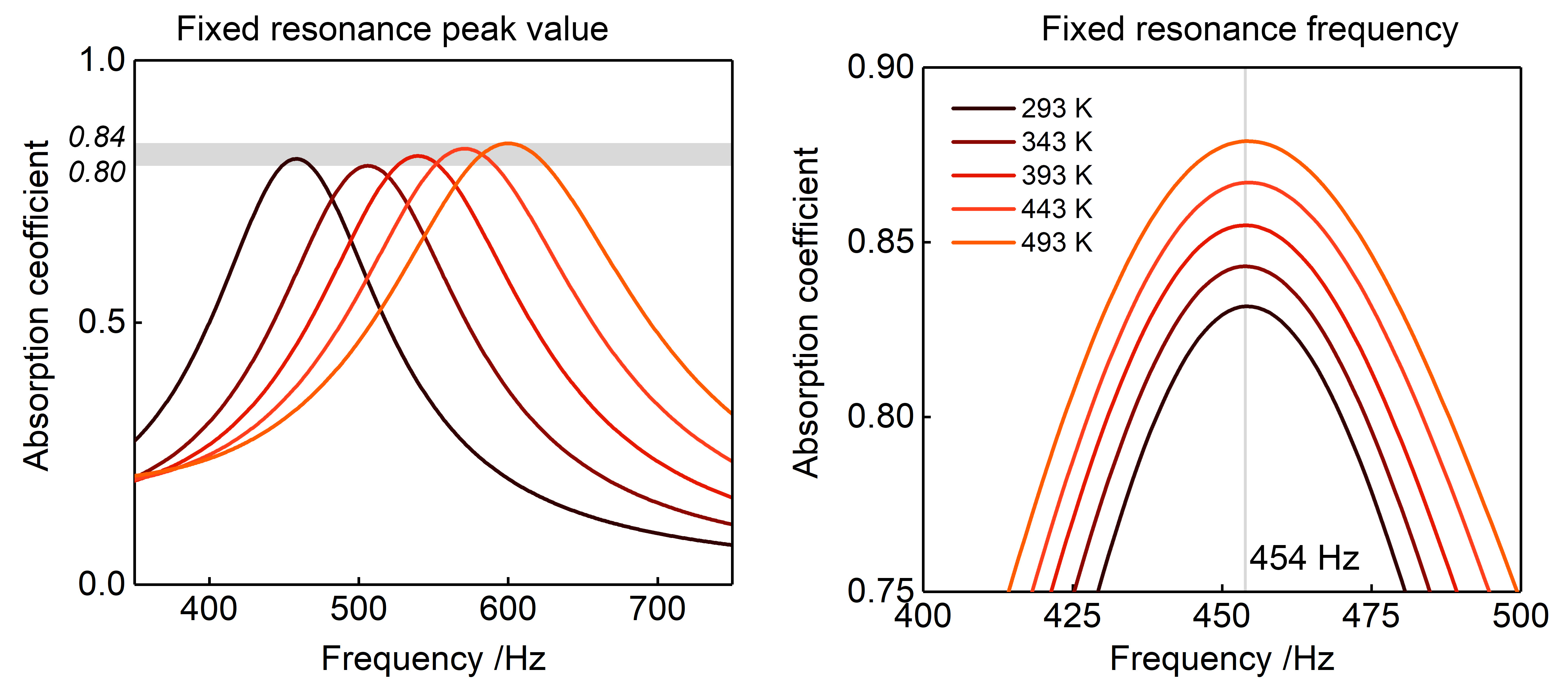


**Figure S5.** Demonstration of geometry-assisted temperature regulation strategies for an under-damped acoustic unit in Fig. 3 (main text). Left: fixed-amplitude frequency modulation, where the absorption peak magnitude is maintained approximately constant while the resonance frequency shifts with temperature. Right: fixed-frequency amplitude modulation, where the resonance frequency is stabilized while the absorption amplitude varies with temperature. These results illustrate that resonance frequency and absorption amplitude are governed by different geometric parameters, enabling cooperative regulation through geometric design and temperature modulation.

## Section S9. Absorption spectra and corresponding impedance characteristics under right‐side incidence


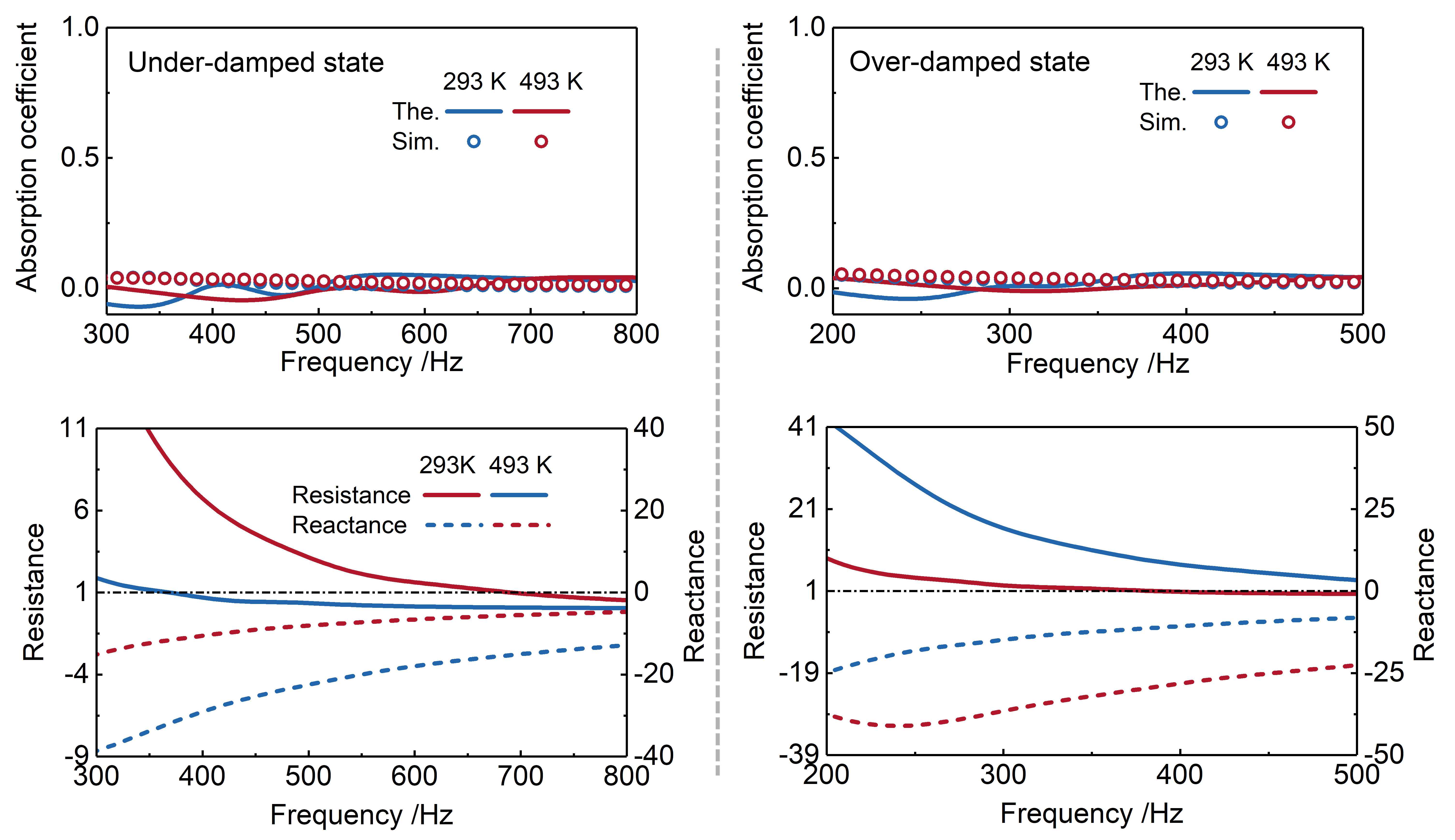


**Figure S6.** Absorption spectra and corresponding impedance characteristics of the coupled TPAM under right‐side incidence for both under‐damped and over‐damped configurations at 293 K and 493 K. Both configurations exhibit nearly zero absorption across the entire frequency range, confirming the pronounced direction-dependent absorption behavior of the loss-asymmetric design.

## Section S10. Summary of superior mechanical and acoustic metamaterials.

**Table S5** compares the proposed metastructure with representative state-of-the-art high-temperature acoustic metamaterial absorber reported in the literature [S8-S11]. Each configuration is evaluated based on key performance metrics, with scoring conducted as objectively and consistently as possible.

|  | This work | Silt-type | Space-coiling | Helical perforation | Porous materials |
| --- | --- | --- | --- | --- | --- |
| Practicability | *High-temperature tolerant, bidirectional two-port absorption. (6/7)* | *Circular-ring slit geometry enables stable sound absorption at elevated temperatures. (6/7)* | *Labyrinthine folded channels enable effective low-frequency absorption in high-temperature environments. (6/7)* | *Helical labyrinthine perforations enable stable low-frequency absorption at elevated temperatures. (5.5/7)* | *Cylindrical perforations in porous media enable robust sound absorption under high-temperature conditions. (6.5/7)* |
| Bandwidth  (Hz) | *Broadband absorption of 321 Hz*  *(5.5/7)* | *Broadband absorption of 362 Hz*  *(6.7/7)* | *Broadband absorption of 172 Hz*  *(4.6/7)* | *Broadband absorption of 207 Hz*  *(4.8/7)* | *Broadband absorption of 460 Hz*  *(6.4/7)* |
| Structural compactness | *A compact structure with a thickness of 40 mm*  *(6.5/7)* | *A compact structure with a thickness of 50 mm*  *(5.5/7)* | *A compact structure with a thickness of 50 mm*  *(5.5/7)* | *A compact structure with a thickness of 50 mm*  *(5.5/7)* | *A compact structure with a thickness of 60 mm*  *(4.5/7)* |
| Tunability | *Frequency/loss tailoring via pores, asymmetry and thermal co-design. (6.5/7)* | *Circular-ring slit dimension. (6/7)* | *folding number and labyrinthine channel geometry. (6/7)* | *Helical diameter and pitch. (6/7)* | *Perforation diameter, spacing, and temperature. (5.5/7)* |
| Fabrication feasibility | *Simple construction of materials (MPP + cavity) enables scaling. (6.5/7)* | *Moderate fabrication complexity due to circular-ring slit machining. (6.5/7)* | *Relatively complex fabrication owing to folded labyrinthine channels. (6/7)* | *Relatively complex fabrication caused by three-dimensional helical perforations. (6/7)* | *Fabrication enabled by straight cylindrical perforations (6.5/7)* |

**Reference**

1. W. Sutherland, *Philos. Mag. J. Sci.* **1893**, 36, 507–531.
2. K. Verdière, R. Panneton, S. Elkoun, T. Dupont, P. Leclaire, *J. Acoust. Soc. Am.* **2013**, 134, 4648–4658.
3. D.Y. Maa, *J. Acoust. Soc. Am.* **1998**, 104, 2861–2866.
4. C. Shen, J. Li, X. Peng, S. Cummer, *Phys. Rev. Mater.* **2018**, 2, 125203.
5. ASTM E2611-17, *ASTM Int.* 2017, West Conshohocken, PA.
6. Y. Zhu, H. Long, C. Liu, H. Zhang, Y. Cheng, X. Liu, *Appl. Phys. Lett.* **2022**, 120, 141701.
7. A. Crivoi, L. Du, Z. Fan, *Appl. Acoust.* **2023**, 205, 109263.
8. W. Yu, J. Ren, Y. Zhang, D. Wang, M. Zhang, B. Song, C. Shen, A. Zhao, *Eng. Struct.* **2025**, 338, 120612.
9. W. Zhang, X. Liu, F. Xin, *Int. J. Mech. Sci.* **2023**, *237*, 107821.
10. W. Zhang, X. Liu, F. Xin, *Mater. Des*. **2023**, 225, 111437.
11. X. Liu, F. Xin, C. Zhang, *J. Appl. Phys.* **2021**, 130, 105101.
